# B cell clonal lineage alterations upon recombinant HIV-1 envelope immunization of rhesus macaques

**DOI:** 10.1371/journal.ppat.1007120

**Published:** 2018-06-22

**Authors:** Christina Yacoob, Miles Darnell Lange, Kristen Cohen, Kanan Lathia, Junli Feng, Jolene Glenn, Sara Carbonetti, Brian Oliver, Vladimir Vigdorovich, David Noah Sather, Leonidas Stamatatos

**Affiliations:** 1 Fred Hutchinson Cancer Research Center, Vaccines and Infectious Diseases Division, Seattle, Washington, United States of America; 2 The Center for Infectious Disease Research, Seattle, Washington, United States of America; 3 University of Washington, Department of Global Health, Seattle, Washington, United States of America; Emory University, UNITED STATES

## Abstract

Broadly neutralizing HIV-1 antibodies (bNAbs) isolated from infected subjects display protective potential in animal models. Their elicitation by immunization is thus highly desirable. The HIV-1 envelope glycoprotein (Env) is the sole viral target of bnAbs, but is also targeted by binding, non-neutralizing antibodies. Env-based immunogens tested so far in various animal species and humans have elicited binding and autologous neutralizing antibodies but not bNAbs (with a few notable exceptions). The underlying reasons for this are not well understood despite intensive efforts to characterize the binding specificities of the elicited antibodies; mostly by employing serologic methodologies and monoclonal antibody isolation and characterization. These approaches provide limited information on the ontogenies and clonal B cell lineages that expand following Env-immunization. Thus, our current understanding on how the expansion of particular B cell lineages by Env may be linked to the development of non-neutralizing antibodies is limited. Here, in addition to serological analysis, we employed high-throughput BCR sequence analysis from the periphery, lymph nodes and bone marrow, as well as B cell- and antibody-isolation and characterization methods, to compare in great detail the B cell and antibody responses elicited in non-human primates by two forms of the clade C HIV Env 426c: one representing the full length extracellular portion of Env while the other lacking the variable domains 1, 2 and 3 and three conserved N-linked glycosylation sites. The two forms were equally immunogenic, but only the latter elicited neutralizing antibodies by stimulating a more restricted expansion of B cells to a narrower set of IGH/IGK/IGL-V genes that represented a small fraction (0.003–0.02%) of total B cells. Our study provides new information on how Env antigenic differences drastically affect the expansion of particular B cell lineages and supports immunogen-design efforts aiming at stimulating the expansion of cells expressing particular B cell receptors.

## Author summary

Broadly neutralizing HIV-1 antibodies (bNAbs) display protective potentials against experimental animal infection and thus are believed to be a key component of an effective HIV vaccine. bNAbs are derived from B cells that express B cell receptors formed by specific VH/VL alleles. We report that the variable domains of recombinant HIV-1 Env immunogens activate a large number of B cell clones that give rise to many non-neutralizing antibodies, and that removing the variable domains from the immunogen reduces the number of activated B cell lineages and leads to the development of autologous neutralizing antibodies, a step towards bNAb-production. Our findings shed new light into how HIV-1 evades detection from B cells that can produce bNAbs and also provides information that is relevant for the design of optimal immunization strategies.

## Introduction

Following HIV-1 infection, serum neutralizing antibody responses against the evolving autologous viral swarm are generated by the vast majority of infected subjects, usually within the first few months of infection [1–6]. In 10–30% of infected subjects, antibodies capable of neutralizing not only the autologous virus but also heterologous viruses are generated, usually following several years of infection [2, 5, 7–13]. These neutralizing antibodies are referred to as broadly neutralizing antibodies (bNAbs). Binding but non-neutralizing antibodies (nNAbs) are also present in sera from infected subjects. Broadly neutralizing monoclonal antibodies isolated from HIV-1-infected subjects protect animals from experimental infection [14–23] and thus bNAbs are expected to be an important component of the protective immune response elicited by an effective HIV-1 vaccine. The viral envelope glycoprotein (Env) is the target for both nNAbs and bNAbs and the epitopes targeted by bNAbs and nNAbs have been identified and in many cases they have been structurally characterized [24–26]. In general, nNAbs target elements of Env that are variable in sequence and are located within the more exposed regions of Env on soluble gp120s or non-stabilized soluble gp140 proteins. In contrast, bNAbs bind conserved elements of Env.

Here we examined whether the variable regions of Env stimulate the expansion of B cell lineages that differ from those expanded by more conserved Env regions, and whether such a differential B cell clonal expansion is linked to the elicitation, or not, of neutralizing antibodies. To this end, we performed immunizations studies in rhesus macaques, since they express IGH, IGK, and IGL V alleles with more than 93% homology to human alleles [27, 28], using two forms of a clade C Env (426c) whose designs we previously described [29–31]: the full length extracellular form (WT) and one where the variable immunodominant regions 1, 2, 3 (V1, V2 and V3, respectively) and three N-linked glycosylation sites (NLGS) were artificially eliminated (‘NLGS-3 Core’). We performed an in-depth analysis of serum antibodies and of isolated monoclonal antibodies as well as IGH/IGK/IGL deep sequencing analyses of the evolving immune B cell responses in the periphery, lymph nodes, and bone marrow.

Although similar serum binding antibody titers were elicited by the two immunogens, the full-length immunogen activated a larger number of B cell clonal lineages than the NLGS-3 Core immunogen. Autologous neutralizing antibodies were elicited only by the NLGS-3 Core immunogen. Binding but non-neutralizing antibodies were derived from B cell clones that became predominant in the periphery, lymph node, and bone marrow during immunization, while neutralizing antibodies were derived from infrequent B cell clonal lineages (0.003–0.02% of total B cells). Our study provides a mechanistic explanation as to how the variable regions of Env elicit high titers of non-neutralizing antibodies. As such, our results support efforts to alter the immunogenicity of non-neutralizing epitopes located in these regions. Furthermore, our approach can be used by others to assess how specific Env modifications alter the activation of particular B cell lineages, or how different adjuvant formulations may alter the activation and expansion of particular unmutated B cell receptors by a particular Env.

## Results and discussion

### The WT and Core immunogens elicit similar serum antibody titers with different epitope specificities

**Fig 1:**
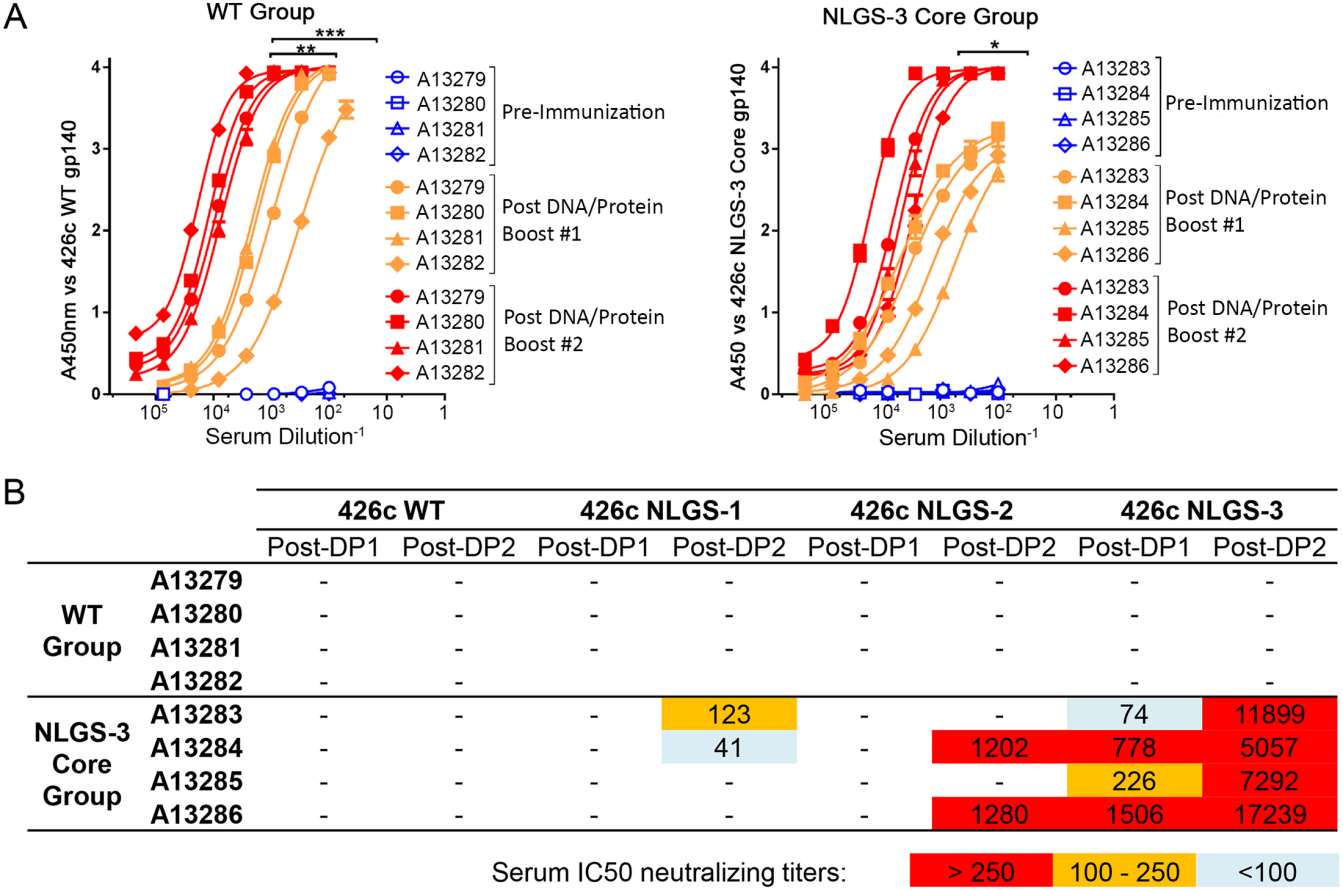
Binding and neutralizing serum activities. (A) Sera collected from the WT *(left)* and NLGS-3 Core *(right)* prior to immunization *(blue)*, following the DNA/Protein Boost 1 *(orange)*, and following the DNA/Protein Boost 2 *(red)* were tested for antibody reactivity to autologous rEnv immunogens. * *indicates p value < 0*.*01*, ** *p value < 0*.*001*, or *** *p value < 0*.*0001*. (B) Reciprocal IC50 serum neutralization titers following the DNA/Protein Boost 1 immunization (Post-DP1) and the Post DNA/Protein Boost 2 immunization (Post-DP2). Neutralizing activities were determined against viruses expressing WT 426c envelope and 426c envelope NLGS variants with mutations in Loop D and V5. NLGS-1 lacks the NLGS at position 276 in Loop D, NLGS-2 lacks two NLGS in V5 at positions 460 and 463, while NLGS-3 lacks all three NLGS.

The immunization schedule and timing of sample collection are summarized in S1 Fig and details are presented in the Materials and Methods section. High titers of autologous binding antibody responses were generated by all animals in both immunized groups, ranging from 7568 to 11924 reciprocal EC50 for the WT immunization group and from 3036 to 7824 for the NLGS-3 Core immunization group (Fig 1A). The titers after the final immunization were not significantly different between the two immunization groups.

In both immunization groups, minimal antibody responses against the gp41 subunit were observed, an indication that the elicited binding antibody responses targeted the gp120 subunits of the immunogens used here. A sizable fraction of the antibodies elicited by the NLGS-3 Core immunized animals targeted the CD4bs, something that was not observed with the WT immunized animals (S2 Fig). Thus, the majority of the serum antibody responses elicited by the WT immunogen recognize epitopes outside the CD4bs.

### Only the NLGS-3 Core immunogen elicits autologous neutralizing antibodies

Serum antibody neutralizing activities were determined following the first and second DNA/Protein (DP) booster immunizations against the autologous 426c WT virus and three NLGS derivatives: NLGS-1: lacking the NLGS in loop D (N276); NLGS-2: lacking the two NLGS in V5 (N460 and N463); and NLGS-3: lacking all three NLGS. We note that the 426c Core Env (i.e., lacking the 3 NLGS and the variable regions 1–3) is not functional and cannot be tested as a virus. Therefore, all four autologous viruses used here express the variable regions 1–3.

The 426c WT virus and these three NLGS derivatives exhibit a tier 2 neutralization phenotype when assayed with sera from chronically HIV-1-infected individuals. As additional evidence of a tier 2 phenotype, all these viruses resisted neutralization by a panel of monoclonal antibodies against the gp120 V3 loop (2219, 2557, 3074, 3869, 447-52D and 838-12D) and CD4bs (654-30D, 1008-30D, 1570D, 729-30D and F105) that are relatively specific for tier 1 viruses. We do want to emphasize that, although the three NLGS-lacking viral derivatives of the 426c virus display tier 2 overall neutralization phenotypes, they are more susceptible to neutralization by certain VRC01-class MAbs than the WT 426c virus.

Irrespective of the immunogen used, neutralization of the WT 426c virus was not recorded (Fig 1B). Only the NLGS-3 Core immunogen elicited serum neutralizing antibody responses against all three autologous NLGS viral variants (Fig 1B). The strongest neutralizing activities (reciprocal IC_50s_) were observed against NLGS-3 and the weakest against NLGS-1. Although 4/4 animals generated anti-NLGS-3 neutralizing antibody responses, only 2/4 animals (A13284 and A13286) generated neutralizing antibody responses against NLGS-2 (and only following the last immunization). Anti-NLGS-1 neutralizing antibodies were also elicited by 2/4 animals (A13283 and A13284), only one of which (A13284) elicited anti-NLGS-2 neutralizing antibody responses. Overall, while 4/4 animals generated anti-NLGS-3 neutralizing antibody responses, one animal (A13284) generated neutralizing antibodies against all three viruses. Interestingly, in the case of animal A13283, vaccine-elicited antibodies neutralized the virus expressing an Env that lacked the NLGS in V5 (NLGS-1), but not the virus that lacked the NLGS in Loop D (NLGS-2). Neutralizing activities against the NLGS-1 and NLGS-2 viruses were not detected following the first DNA / Protein immunization.

Thus, in animals immunized by the NLGS-3 Core immunogen, only a fraction (Log differences) of neutralizing antibodies can bypass the glycan steric blocks presented on Loop D and/or V5, but not both (i.e., the WT Env). Overall, the results suggest that: (a) the NLGS-3 Core immunogen can elicit autologous NAbs that can ‘by-pass’ the variable regions 1, 2 and 3 (which are absent from the immunogen, but present on the virus), as long as the NLGS in V5 or Loop D are absent (individually or in combination); (b) that these NAbs have a harder time ‘bypassing’ the NLGS in V5 (N460 and N463; i.e., the NLGS-1 virus) than the NLGS in loop D (N276; i.e., the NLGS-2 virus); and (c) that the autologous NAbs elicited by the NLGS-3 Core target an epitope whose relative exposure on the virus is regulated by the presence of NLGS in Loop D and V5, similar to what is known for several anti-CD4bs bNAbs [31–35].

The sera did not display neutralizing activity against several heterologous viruses (Clade A: Q168a2, Q461e2, Clade B: QHO692, SF162, Bal.26, and Clade C: 706c, 823c). Elimination of the NLGS (in Loop D and V5) from most of these viruses (Q168a2, Q461e2, Bal.26, 706c and 823) did not lead to neutralization either. Thus, the neutralizing antibodies elicited by the NLGS-3 Core in non-human primates (NHPs) using this immunization regimen target epitopes that are either absent or are present but are less accessible on heterologous viruses.

### Narrow IGH/IGK/IGL-V stimulation by the 426c Core NLGS-3 immunogen

To determine whether the different abilities of these two immunogens to elicit neutralizing antibody responses were linked to a differential stimulation of B cell lineages by the two immunogens, we performed next generation Illumina MiSeq deep sequencing analysis of the variable domains of the heavy (IGHV) and light (IGKV and IGLV) chains from B cells isolated from the periphery, pre- and post-immunization.

**Fig 2:**
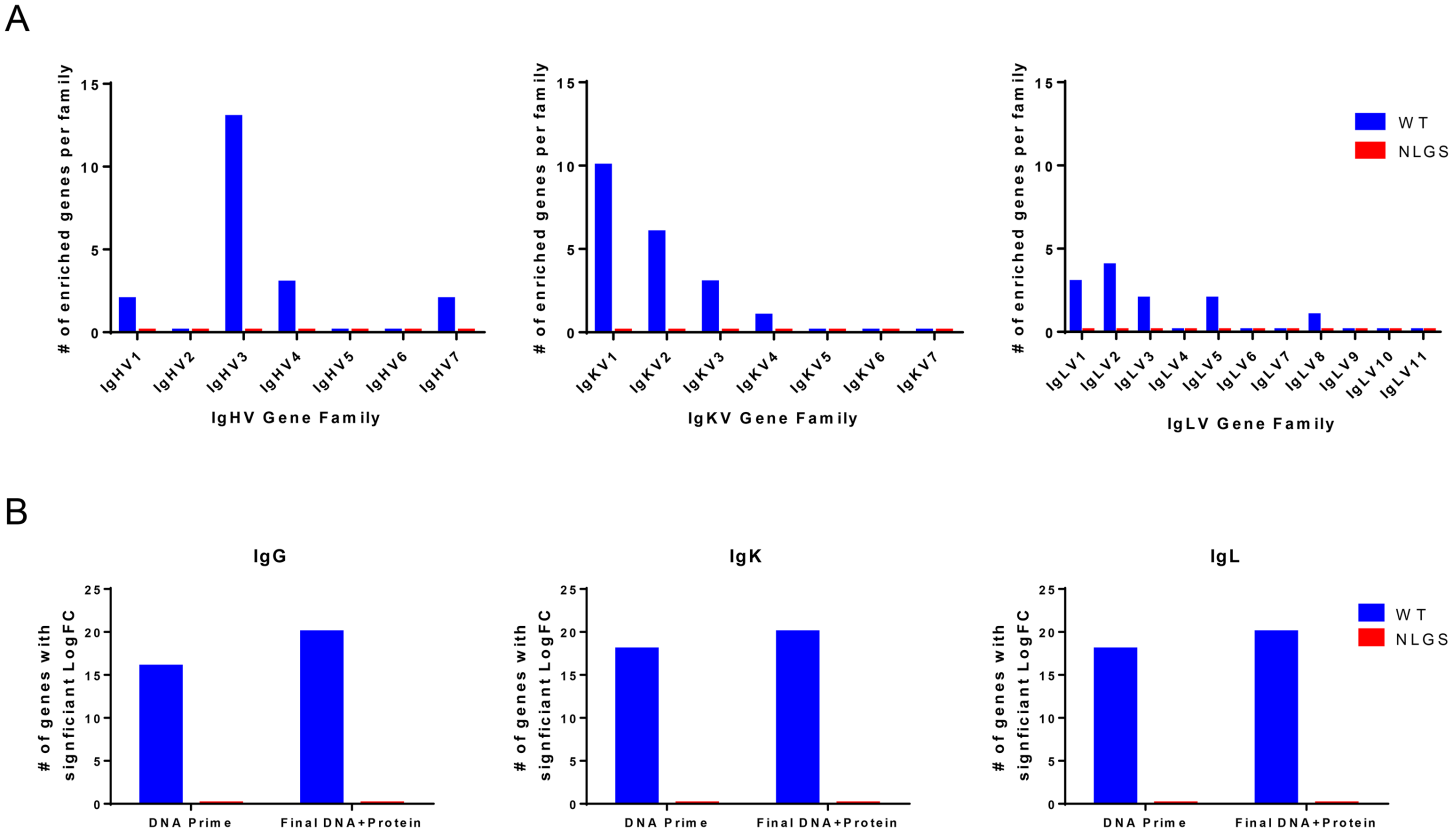
Changes in IGH, IGK and IGL frequencies upon WT and NLGS-3 Core immunization. (A) Number of genes in each V gene family with a significant positive Log Fold Change (LogFC) value for WT (*blue*) and NLGS-3 Core (*red*) immunization groups. Positive LogFC was calculated and compiled between the pre-immunization and post DNA/Protein Boost 2 for IgHV *(left)*, IgKV *(middle)*, and IgLV *(right)* gene families. All LogFC have a false discovery rate of <0.05. (B) Total number of V genes with a positive LogFC and FDR < 0.05 for WT and NLGS-3 Core immunization groups between pre-immunization and post DNA Prime or post DNA/Protein Boost 2 for IgHV *(left)*, IgKV *(middle)*, and IgLV *(right)* V alleles. Bars in A and B represent the total number of genes with significant LogFC. Bars at baseline indicate no genes scored a significant LogFC.

A number of IgHV genes from circulating memory B cells became commonly enriched among the animals from the group immunized with WT 426c Env. In the IGH locus, genes belonging to the IGHV3 family underwent the most expansion (13 total genes), and to a much lesser extent we observed expansion in the IGHV1 (2 genes), IGHV4 (3 genes) and IGHV7 (2 genes) (Fig 2A, S3 Fig Top panel). Stimulation of these genes was observed after both DNA immunization and after protein plus DNA immunization (Fig 2B). This, despite the fact that IGHV1 represents <5% and IGHV3 ~20% of circulating IGHV in IgM^+^ B cells in NHP, while IGV4 is the most frequently expressed IGVH (~70%) in IgM+ B cells [28]. However, we previously reported [28] that the IgGHV3 family becomes more prevalent in the IgG^+^ B cell compartment compared to the IgM^+^ compartment, indicating a preferential stimulation of IGHV3 into class-switched memory B cells, in agreement with what we observed here. The particular expansion of the above-mentioned VH genes was also reported by others in studies were a different full length soluble Env was used [36, 37]. Thus, it is probable that the stimulation of IGHV1 and IGHV3 of the circulating memory B cells is due to immunization with Env.

In the light chain loci, we observed enrichment in both the IgK and IgL loci after immunization with WT 426c (Fig 2A). In the IgK locus, IgKV1 was the most enriched, followed by IgKV2, IgKV3, and IgKV4. In the IgL locus, the IgLV2 family was the most enriched after immunization, followed by IgL1, IgLV3, IgLV5, and IgLV8 families. As with the IgH locus, stimulation of the light chain families was observed after both DNA and DNA plus protein immunization (Fig 2B). IgKV1 and IgLV2 are the predominantly expressed gene families from their respective loci [28].

We did not observe any significantly enriched IGHV, IGKV, or IGLV gene families after immunization with NLGS-3 Core (Fig 2), indicating that there was not a widespread stimulation of the same V genes within the group. We determined that this finding was not due to the NGS sequence data sets themselves, as quality and Hill’s diversity analysis of all sequence sets reported here revealed all data sets to be roughly equivalent in structure and quality, no matter the chain that was amplified nor the origin of the libraries (S4–S7 Figs) [38]. These findings were confirmed by principal component analyses, which clusters large, multi-dimensional data sets by the most significant sources of variation. In the WT animals, the NGS data sets clustered by time point, indicating that the statistically significant changes in gene abundance were due to vaccination time point. In contrast, the NLGS-3 NGS data sets cluster by animal and not time point, confirming that vaccination did not drive significant changes in common gene usage among the animals in this group (S8 Fig). This stark dichotomy implies that, while the NLGS-3 is immunogenic and elicits IgG titers similar to that of WT 426c, it does not broadly stimulate a diversity of V genes during immunization. Potentially, this is a direct, measurable consequence of the elimination of the highly immunogenic variable loops.

### Epitope-specificity of B cells producing neutralizing antibodies

**Fig 3:**
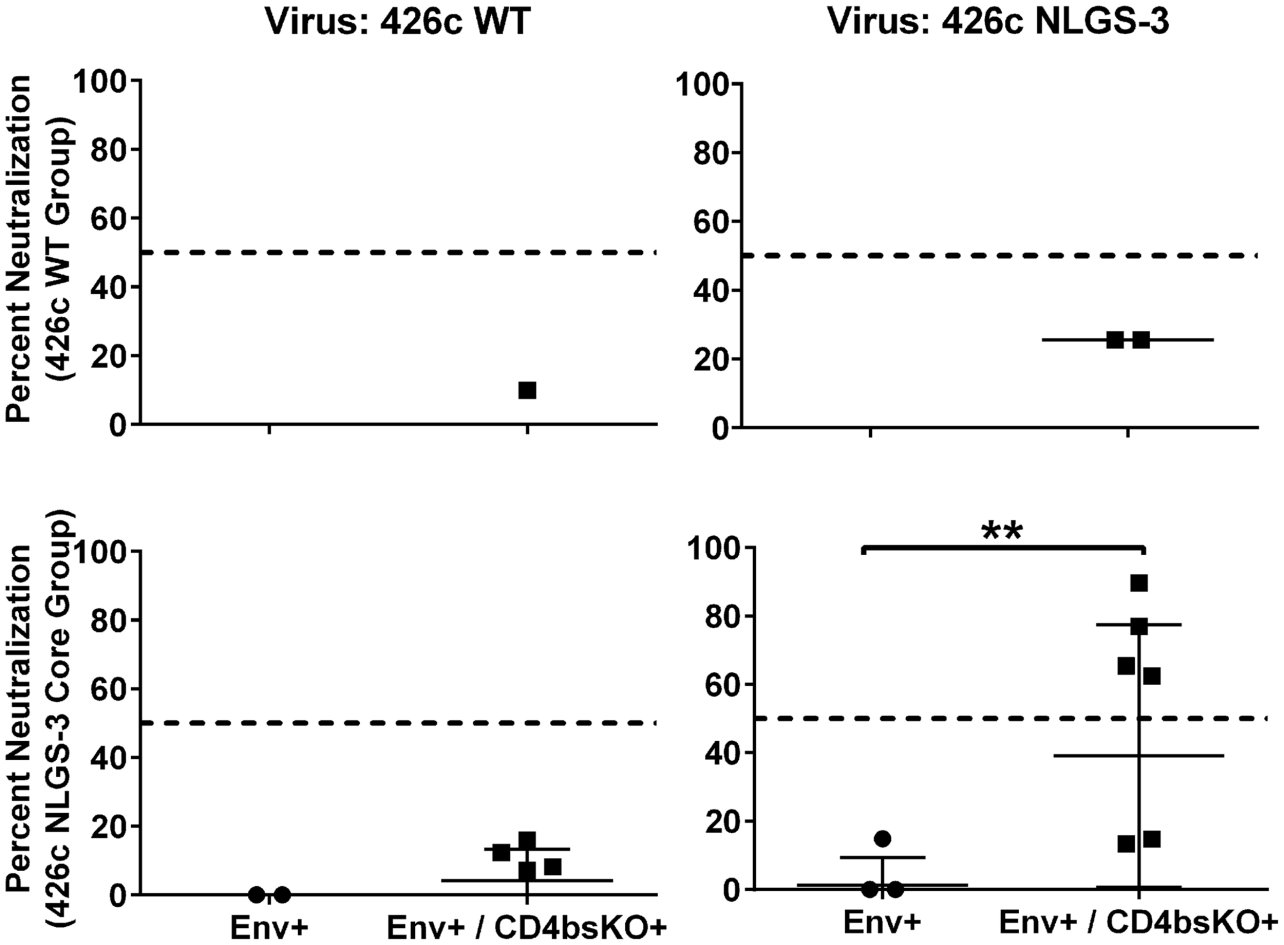
*Ex vivo* production of neutralizing antibodies from B cells isolated animals immunized with the NLGS-3 Core immunogen. IgG+ B cells were sorted from PBMC and cultured as discussed in the Materials and Methods section. B cell supernatants were tested for neutralizing activity against the WT 426c virus (426c WT; left panels) or a variant lacking three NLGS at positions N276, N460 and N463 (426c NLGS-3; right panels). Results from supernatants from B cells isolated from animals immunized with the WT immunogen are shown in the top two panels, while results from the NLGS-3 Core immunized animals are shown in the bottom two panels. Each dot corresponds to a single well (which contained approximately 1,000 B cells).

To better characterize the B cells that produce neutralizing antibodies and those that produce binding but not neutralizing antibodies, we isolated Env-specific IgG B cells from individual animals following immunization based on their CD4bs specificity (based on the D368R and E370A mutations, DREA). Thus, two populations of B cells were isolated from animals immunized with either immunogen: CD4bs-specific cells (Env^+^/CD4bs-KO^-^ B) cells and non-CD4bs-specific cells (Env^+^/CD4bs-KO^+^ B cells). The corresponding recombinant Env used to immunize the animals was used for B cell-isolation. B cells were cultured in bulk in multiple wells, each well containing ~1000 B cells, due to the high number of sorted B cells. The cell supernatants were evaluated for anti-WT 426c and anti-NLGS-3 virus neutralizing activities (Fig 3). Supernatants from wells containing B cells (irrespective of their CD4bs specificities) isolated from the WT-immunized animals did not display neutralizing activities. In contrast, supernatants from 4 of 6 wells containing non-CD4bs specific B cells isolated from the NLGS-3 Core-immunized animals neutralized the autologous NLGS-3 virus, but not the WT virus. Thus, the neutralization results obtained from B cell supernatants and those obtained from sera (Fig 1B) were in agreement. The NLGS-3 neutralizing activity was derived from non-CD4bs-specific B cells. Since these B cells were isolated from animals immunized with the NLGS-3 Core immunogen, by definition they do not bind elements of V1, V2, or V3 regions. Thus, although the majority of serum binding antibodies target the CD4bs (S2B Fig), the neutralizing activity in the sera is due to antibodies whose binding is independent of the DREA mutation that is widely used to identify anti-CD4bs antibodies in sera [5, 39–41].

### Paired IGH and IGK/IGL amplification and sequencing from individual B cells

To better define the characteristics of the neutralizing antibodies elicited by NLGS-3 Core and to compare them to those of non-neutralizing antibodies elicited by the same immunogen, we isolated individual CD4bs-specific and non-CD4bs-specific and peripheral IgG+ B cells from the four animals immunized with the NLGS-3 Core (S9 Fig). The CD4bs-specific B cells represented the minority of Env-specific B cells (between 4.5% and 8.6% of total Env^+^ B cells. Within the non-CD4bs-specific B cell population, the neutralizing B cells represented a small fraction (between 0.4% and 2.4%). Thus, only a very small fraction (0.003 to 0.02%) of total periphery IgG+ B cells display neutralization potential.

IGH and IGK/IGL genes from wells containing individual B cells that displayed neutralizing activities against the NLGS-3 virus and from wells displaying 426c NLGS-3 Core-binding, but not neutralizing activity were amplified and sequenced. Seventy-nine IGH (S10 Fig) and 32 IGK/IGL (S11 Fig) genes were fully sequenced from peripheral B cells secreting binding, but not-neutralizing antibodies. The majority of IGH (~90%) were derived from IGHV3 and IGHV4 alleles. The majority of the IGHV3 sequences (~52%) were derived from the IGHV3-Korf19 allele (human homologue is IGHV3-33*01 [28]). A majority of the IGK light chains were derived from the IGKV1 family (~28%) (S11 Fig), including IGKV1-I21 (human homologue IGKV1D-16*01) which represented 10.9% of IGK present in the peripheral B cells. The second most frequently expressed LC was derived from IGLV5. Within the IGLV5, the predominant allele was IGLV5-S28. Overall, we estimate that over 40% of the serum response was represented by these binding, but non-neutralizing antibodies, due to their allelic dominance in the peripheral B cell repertoire.

#### Neutralizing activities of MAbs

Twenty-six wells displayed neutralizing activity against the NLGS-3 virus. We successfully amplified 14 paired HC/LC sequences (S12 Fig). Of these, 2 were unique (SB0409 from animal 13283 and SB0206 from animal 13286) and 6 were clonally related (from animal 13284), which were derived from IGHV2-Korf1 whose human homologue is IGHV2-70D*04) [28]. The neutralizing MAbs displayed a range of CDRH3 (12–16) and CDRL3 (8–11) amino acid lengths and ~2–9% nucleotide somatic hypermutation (S13 Fig, S1 Table). The IGHV3-FI*01 allele, from which MAb SB0409 was derived from, was found in relatively high average frequencies in the NGS data sets from the periphery (12.5%), LN (9.0%) and bone marrow (22.6%) of NLGS-3 Core immunized animals while the IGHV of the other MAbs were very infrequent or undetectable. All six clonally related neutralizing antibodies expressed the IGKV1-S37 light chain (whose human homolog is IGKV1D-16*01), which is expressed at 9.5% in the NGS data sets from the periphery. The remaining two light chains were IGKV2-IS4 (human homologue IGKV2-30*02) and IGLV3-S46 (human homologue IGLV3-21*01) and were minimally detected in peripheral B cells (0.6% and 0.03%, respectively).

**Fig 4:**
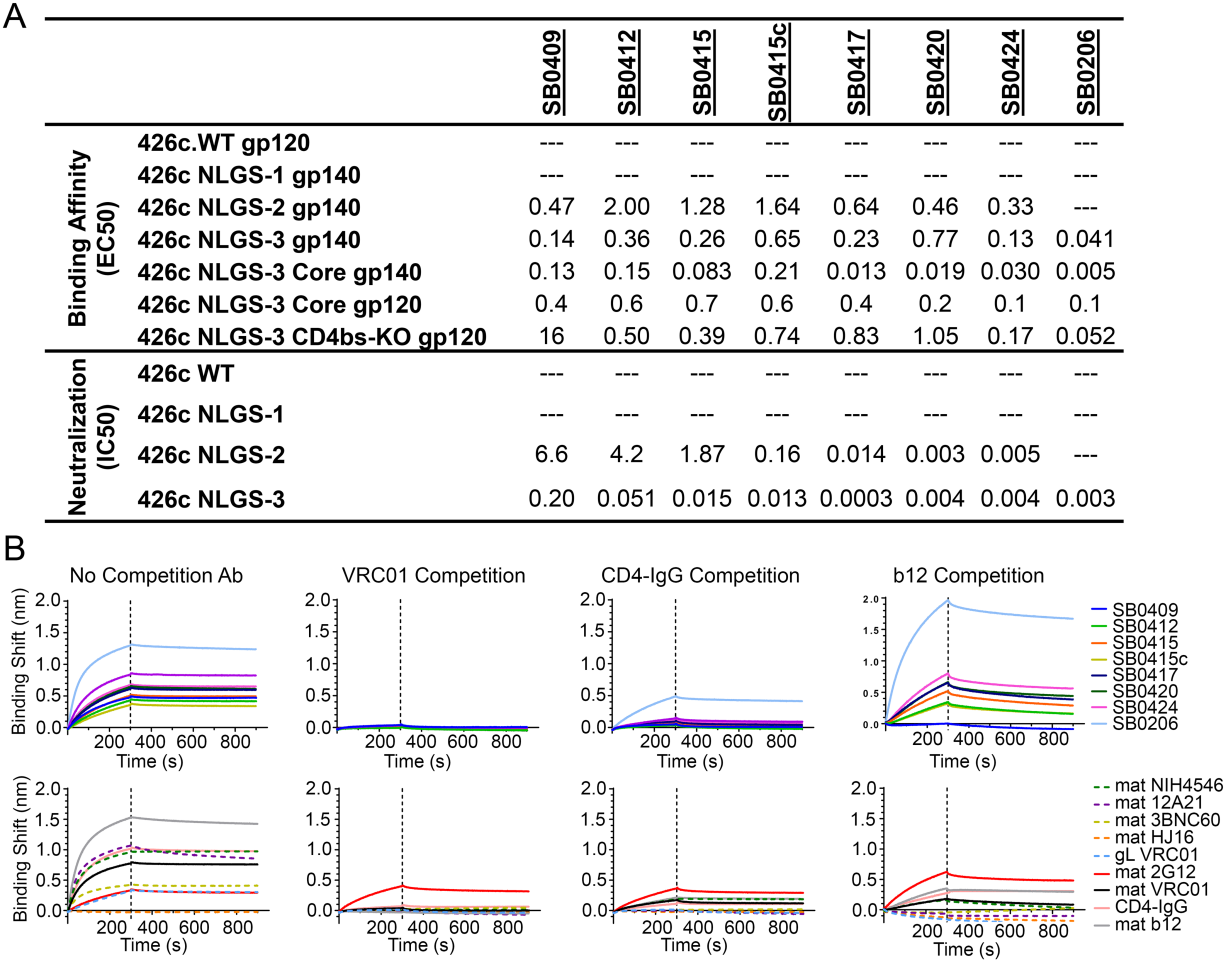
Binding and neutralizing potentials of MAbs isolated from the NLGS-3 Core immunized animals. (A) Binding (*top*) and neutralizing (*bottom*) titers of the indicated macaque MAbs against the indicated Envs and viruses. (B) Antibody-binding competition. Top panels: The binding of the indicated macaque MAbs to the 426c NLGS-3 Core gp140 protein was determined in the absence of competing antibody *(left)*, or following the pre-incubation of 426c NLGS-3 Core gp140 with saturating concentrations of VRC01 *(middle left)*, CD4-IgG *(middle right)*, or b12 *(right)*. Bottom panels: Control experiments were performed with the indicated human MAbs. Direct binding of the indicated human MAbs to the 426c NLGS-3 Core gp140 protein (*left panel*), following the pre-incubation of Env with VRC01 (*second from left panel*), CD4-IgG (*second from right panel*), or b12 (*right panel*).

In agreement with the serum neutralization results (Fig 1B), the MAbs did not neutralize the 426c WT virus, or the variant lacking N276 (NLGS-1) (Fig 4A). Also in agreement with the serological results, MAb SB0409 that was isolated from animal 13283 did not neutralize the NLGS-2 virus, while 5/6 MAbs isolated from animal 13284 did neutralize this virus and all MAbs neutralized the NLGS-3 virus. In contrast, however to the serological results, the MAbs isolated from animals 13283 and 13284 did not display anti-NLGS-1 virus neutralizing activities. We note that the anti-NLGS-1 serum neutralizing activities in these two animals were low (Fig 1B) and we expect the frequency of B cells expressing such antibodies to be low, thus rendering their isolation difficult.

#### Epitope Mapping of MAbs

In agreement with the absence of neutralization activities against the WT and NLGS-1 viruses, the 8 MAbs did not display reactivities against the corresponding gp140s and did not neutralize the corresponding viruses (Fig 4A). With the exception of MAb SB0206, the MAbs bound the NLGS-2 and NLGS-3 gp140s and neutralized the corresponding viruses (Fig 4A). The binding of these MAbs to the NLGS-3 Env that also lacks the variable regions 1, 2 and 3 (NLG2-3 Core) were greater than towards the NLGS-3 Env, suggesting that the variable loops encumber the access of these MAbs to their epitopes. These MAbs also bound to the NLGS-3 DREA Env (CD4bs-KO), but with much lower binding affinities than to the NLGS-3 Env. This observation and the fact that these MAbs were isolated from B cells that bound to both NLGS-3 and NLGS-3 DREA probes, suggests that although the DREA mutations do not abrogate their binding completely, they do reduce it. It is thus plausible that these MAbs recognize epitopes within or close to the CD4bs. In support of this, their binding was abrogated when Env was pre-incubated by the anti-CD4bs bNAb VRC01 (Fig 4B) and in 7/8 cases by IgG-CD4 (the binding of MAb SB0206 was reduced but not abrogated). Also, in 7/8 cases the binding was reduced when Env was pre-incubated by a different CD4bs antibody, b12 (the binding of MAb SB0409 was actually abrogated). The differential effect of the DREA mutations on the binding of these MAbs suggests that these MAbs recognize the same overall gp120 region but with different angles of approach.

## Discussion

Different concepts to elicit HIV-1 bNAbs through immunization are under investigation. One concept is based on the ‘germline-targeting’ approach during which a ‘germline-targeting’ immunogen is used to initiate the activation of naïve B cells expressing specific germline (unmutated) BCRs and subsequently, booster immunizations with specifically-designed immunogens to guide the maturation (through somatic hypermutation) of these BCRs towards their broadly neutralizing forms [42].

This approach was recently shown to be effective in eliciting PGT121 bNAbs in a knock-in mouse model where the PGT121 germline BCR was expressed by every B cell [43]. On a polyclonal BCR background however, Env immunogens (including ‘germline-targeting’ immunogens) will activate not only the desired B cells, but many B cells that recognize irrelevant, but immunogenic, epitopes on the immunogen [44]. These off-target B cells will expand even further during the booster immunizations, potentially limiting the expansion of the desired B cells (although this has not yet been experimentally demonstrated). We (and others) believe that an in-depth understanding of BCR clonal lineages expanding during immunization with Env-based immunogens will be important to identify the booster immunogens for the optimal development of bNAbs [45].

The NLGS-3 Core Env immunogen used here, was engineered by introducing specific mutations on the tier 2 clade C 426c viral Env. These include deletions of the variable domains 1, 2 and 3 and the targeted elimination of three NLGS: one in Loop D (N276) and two in V5 (N460 and N463) [29, 31, 46]. These modifications were introduced so that this Env engages B cells expressing germline VRC01-class BCRs, which are derived from the human VH1-2*02 allele paired with LCs expressing infrequent 5 amino acid long CDRL3 domains.

We now know that rhesus macaques (and other animal species such as mice, rats and rabbits) do no express an exact orthologue to the human VH1-2*02 allele [28, 47, 48]. The present study was not therefore conducted to inform on the ability of the NLGS-3 Core immunogen to activate and expand B cells expressing germline VRC01-like BCRs *in vivo*, but to generate new mechanistic information on why the variable V1, V2 and V3 domains of Env dominate the B cell responses upon Env immunization. In this regard we note that although the observed relative changes in IGH and IGK/IGL were assessed from ‘total’ B cells and not exclusively from Env-specific B cells, we expect that these changes are due to differences in the antigenic/immunogenic properties of the two immunogens we evaluated here, as the immunogens were prepared (expressed and purified) identically, the same adjuvant was used and the immunization protocols were identical. We also note that although the NGS analyses of IGH and IGK/IGL were performed on the same samples, they were derived from bulk, but not individual B cells and thus presently we cannot assess whether the expansion of a particular IGH lineage was linked with the expansion of a particular IGK/IGL lineage.

At first glance, it is not surprising that the WT immunogen stimulated a larger number of IGH and IGK/IGL lineages, as it expresses more epitopes than the NLGS-3 Core; especially the variable V1, V2 and V3 domains which are known to be immunogenic, both in the context of HIV-1 infection and immunization with Env [39, 40, 49–55]. One would expect that the immunogenicity of epitopes present on the core part of Env to increase in the absence of the immunodominant variable domains. This was the case, as the NLGS-Core elicited similar serum antibody titer responses as the WT immunogen.

We note that both immunogens examined here are not stabilized trimers. It is anticipated that the immunogenicity of variable domains will be reduced on such constructs [56–59]. The fact however, that neutralizing antibodies against the autologous NLGS viruses were elicited by the immunogen lacking the variable regions 1, 2 and 3, indicate that neutralization epitopes are located outside these variable regions of Env; within the core components of Env. These epitopes are also present in the WT immunogen (as the two immunogens share a common amino acid sequence), but since that immunogen did not elicit neutralizing antibodies we assume that these epitopes are occluded and thus poorly immunogenic. The lack of neutralization of the WT 426c virus by the neutralizing antibodies elicited by the NLGS-3 Core immunogen supports this assumption. In part the occlusion of the epitopes is due to carbohydrates present on NLGS in Loop D and V3, as viruses lacking these NLGS are susceptible to the neutralizing antibodies elicited by the NLGS-3 Core immunogen.

One (13284) of four animals immunized with NLGS-3 Core elicited serum neutralizing antibody responses against both the NLGS-2 virus (lacking the two NLGS in V5; positions N460 and N463) and the NLGS-1 virus (lacking the NLGS in Loop D; position N276). The neutralization titers against the NLGS-2 virus were 30 fold higher than those against the NLGS-1 virus. This, combined with the fact that none of the isolated neutralizing antibodies neutralized the NLGS-1 virus, but 5/8 neutralized the NLGS-2 virus, suggests that B cells producing antibodies capable of bypassing the restrictions imposed by the V5 NLGS were less frequently expanded during immunization than the B cells producing antibodies capable of bypassing the Loop D NLGS. As the conserved NLGS in Loop D (N276) is a major block in the engagement of germline VRC01-class BCRs by Env [31–33, 35, 47, 60, 61], our results suggest that the 426c NGLS-3 Core immunogen presents CD4bs epitopes more favorably as compared to the WT Env even in animals without VRC01-like naïve B cells. An alternative possibility is that the NLGS-3 Core mutations themselves are the cause of the development of the neutralizing antibody responses discussed here. As we did not immunize animals with a 426c Env that only lacked the 3 NLGS, we do not know the relative impact these NLGS or the variable regions had on the observed B cell lineage expansions observed with the 426c NLGS-3 Core immunogen.

Regardless, our results suggest that in animals with a polyclonal naïve BCR repertoire capable of producing VRC01-class B cells immunization with the 426c NGLS-3 core immunogen may lead to activation of VRC01-like naïve B cells.

Our underline hypothesis for the observations made here, is that the epitopes targeted by the neutralizing antibodies elicited by the NLGS-3 Core immunogen are occluded on the 426c WT Env and thus are not immunogenic. It is however possible that they equally immunogenic on the 426c WT Env, but because the immunogenicity of the variable domains is so high, it overwhelms the response to the neutralizing epitopes. We previously reported that non-neutralizing anti-CD4BS antibodies can prevent the uptake of Env by B cells expressing precursors of broadly neutralizing antibodies ([29]). The outcome of the competition between ‘on target’ and ‘off target’ B cells responses to Env depends on the relative frequencies of ‘on-target’ and of ‘off-target’ B cells (that are always going to be present at some level) and on the relative affinities of the ‘on-target’ and ‘off-target’ BCRs to their respective epitopes on the same immunogen ([62, 63]). Thus, immunogen-design approaches that aim at reducing the immunogenicity of non-neutralizing epitopes on Env immunogen and at increasing the affinity of BCRs for neutralizing epitopes are fully warranted.

## Materials and methods

### Recombinant envelope proteins

All Env constructs are based on the HIV-1 clade C 426c Env (GenBank: KC769518.1). Mutations that disrupt N-linked glycosylation sites (NLGS) were introduced, individually and in combination, in Loop D (N276) and V5 (N460 and N463) to generate the following single, double and triple mutants: N276D (NLGS-1), N460D+N463D (NLGS-2), and N276D+N460D+N463D (NLGS-3) [31]. Deletions of the variable regions 1, 2, and 3 were also introduced on the NLGS-3 background (this construct is referred to as ‘NLGS-3 Core’ [46]. D368R/E370A mutations that knock-out the binding of many anti-CD4-binding site antibodies (CD4-binding site KO, CD4bs-KO) were also introduced on some of the above-mentioned constructs. CD4bs-KO reagents were employed during the B cell-sorting experiments (see below). Soluble gp140 or gp120 forms of these Envs were expressed from the pTT3 vector [29, 31].

Soluble recombinant gp120 envelopes produced by transient transfection of 293E/F suspension cells and purified using a size-exclusion chromatography AKTA purifier (GE, Fairfield, CT) as described previously [64, 65]. Avi-tagged versions of WT, NLGS-3 Core, or NLGS-3 Core with CD4bs-KO mutations Envs were biotinylated overnight using the BirA enzyme *in vitro* biotinylation kit (Avidity, Aurora, CO) with an excess of biotin. Excess biotin was removed via Amicon Ultra-4 centrifugal membrane filtration (EMD-Millipore, Billerica, MA, USA). Streptavidin-allophycocyanin (SA-APC) or streptavidin-allophycocyanin-Cy7 (SA-APC-Cy7) was conjugated to biotinylated proteins at an optimized ratio.

### Animals and immunizations

Two groups (four animals each) were immunized with either 426c WT (Animals IDs: A13279, A13280, A13281, and A13282) or 426c NLGS-3 Core (Animals IDs: A13283, A13284, A13285, and A13286) (gp140 forms) (S1 Fig). The latter construct expressed the I423M / N425K / G431E mutations that reduces binding to human and macaque CD4 [66]. At weeks 0 and 4, the animals were immunized with DNA vectors expressing the gp140 Env forms. 2mg DNA in 1mL endotoxin-free water was administered intradermally in 2 sites in the back (0.2mg each) and intramuscularly in 2 sites in the quads (0.8 mg each). Protein immunizations were administered with 20% Adjuplex at weeks 12 and 20. 0.1mg protein in 0.5mL 20% adjuvant mixture was administered intramuscularly in the deltoids. Blood was collected at weeks -4, -2, 1, 2, 5, 6, 12, 13, 14, 20, 21, and 22. Lymph nodes (axillary and/or inguinal) were collected at weeks -2, 13, 21, and 39, and bone marrow collected at week 22.

PBMCs, plasma, and bone marrow were purified from freshly-collected blood using density gradient centrifugation with Ficoll-Paque and SepMate columns (StemCell Technologies, Vancouver, BC, Canada) according to adapted manufacturer’s instructions. Isolated PBMCs were resuspended (20 x 10^6^ cells/mL) in freezing media (90% heat-inactivated FBS, 10% DMSO), placed in Mr. Frosty containers (ThermoFisher Scientific, Waltham, MA), and stored at -80°C overnight before transfer to liquid nitrogen, where they were stored until further use. Plasma was aliquoted and stored at -80°C. Lymph nodes were sliced into grindable parts and cell strained using a 40μm strainer followed by a rinse with RPMI-1640 media, and then a rinse with 10mL PBS (Thermo Fisher Scientific, Waltham, MA). Isolated lymph node cells were resuspended (10 x 10^6^ cells/mL) in freezing media (90% heat-inactivated FBS, 10% DMSO), placed in Mr. Frosty containers, and stored in -80°C overnight before transfer to liquid nitrogen, where they were stored until further use.

### Ethics statement

All NHP studies were conducted at the Washington National Primate Research Center at the University of Washington (Seattle, WA, USA). The study was reviewed and approved by the UW Institutional Animal Care and Use Committee, Office of Animal Welfare, University of Washington under Protocol Number: 3408–04 and Protocol Title: Optimizing HIV Immunogen-BCR Interactions for Vaccine Development. Housing and care procedures were within guidelines of the National Institutes of Health (NIH) (National Research Council, Guide for the Care and Use of Laboratory Animals, 8th edition) and in compliance with federal regulations relating to animal welfare. All efforts were made to minimize suffering. Details of animal welfare and steps taken to ameliorate suffering were in accordance with the recommendations of the Weatherall report, "The use of non-human primates in research". Rhesus macaques (Macaca Mulatta) of Indian origin, approximately 3 years old, were habituated to the housing conditions (> 4 weeks) before the initiation of the study. All procedures were conducted under anesthesia (10mg/kg ketamine HCL). Animals were individually housed in suspended stainless steel wire-bottomed cages and provided with a commercial primate diet. Fresh fruit was provided once daily and water was freely available at all times. A variety of environmental enrichment strategies were employed. The animals were not terminated at the conclusion of study, and were released back into the colony.

### B cell isolation

10–20 million PBMCs, lymph nodes (LNs), or bone marrow (BM) were thawed and resuspended in 12mL complete RPMI (2% Penn-strep, 10% heat-inactivated FBS), centrifuged at 1,400rpm for 5 min, then rinsed with FACS Buffer (2% heat-inactivated FBS in sterile PBS). The cells were resuspended in 1 – 2mL RBC lysis buffer (Sigma, St. Louis, MO) for 10min at RT to lyse red blood cells according to manufacturer’s instructions. 8 – 10mL of 1x PBS was used to rinse cells. The cells were resuspended in 50–100μL PBS, and then stained with 0.5–1μL of Live/Dead Fixable Aqua Dead Cell Stain according to manufacturer’s protocol (Invitrogen/Life Technologies, Grand Island, NY). Cells were then stained with NLGS-3 Core CD4bs-KO–APC-C7 (426c.NLGS-3.D368R.E370A.MKE.DV1/2/3-APC-Cy7) for 10min on ice in the dark, then with either WT- APC or NLGS-3 Core rEnv—APC (426c.WT-APC or 426c.NLGS-3.MKE.DV1/2/3-APC) for 10min. Cells were then stained with a master mix of CD3-FITC, clone SP34 (BD Biosciences, San Jose, CA), CD14-FITC, clone MφP9 (BD Biosciences, San Jose, CA), CD19-PE, clone J3-119 (Beckman Coulter, Brea, CA), and IgG PECF594, clone G18-145 (BD Biosciences, San Jose, CA). For compensation set-up, PBMCs collected prior to immunization were used. IgG-APC, clone G18-145 was utilized for the compensation control in the APC channel due to the minimal binding of the rEnv-APC to naïve rhesus macaque PBMCs (BD Biosciences, San Jose, CA). All samples were resuspended in 1 mL media (~1M cells /mL) and filtered through 70μm Flowmi strainers (Scienceware Bel-Art, Wayne, NJ). LD Aqua^-^ / CD3^-^ / CD14^-^ / CD19^+^ / IgG^+^ / WT^+^ or NLGS-3 Core rEnv^+^ / NLGS-3 Core CD4bs-KO rEnv^+/-^ cells were bulk sorted into 100μL complete IMDM media using a FACS Aria II cell sorter (BD Biosciences, San Jose, CA). Sorted rEnv-specific cells were plated on 3T3-msCD40L feeder cells (provided by Dr. J.R. Mascola, NIH/VRC, 3T3-msCD40L are NIH 3T3 mouse embryonic fibroblast cells engineered to express the CD40 ligand) at a final dilution of 1.4 B cells/well and cultured as previously described [67]. After 12 days, supernatants were collected for ELISA and neutralization (see below) testing and the cells were lysed with 30μL of RLT supplemented with β-ME/glycogen and frozen at -80°C. Prior to single cell sorting, negative and positive populations of WT rEnv^+^ / NLGS-3 Core rEnv^+^ and NLGS-3 Core CD4bs-KO rEnv^+/-^, cells were sorted into 100μL of complete IMDM media supplemented with 2% Penn-strep and 10% heat-inactivated FBS then plated at 1000 cells/well on 12-well plates with 3T3-msCD40L feeder cells with conditions described above. After 12 days, supernatants were removed and were tested for neutralizing activity (see below) and the cells were lysed with RLT supplemented with β-ME/glycogen and stored at -80C.

### Heavy and light chain sequencing from single cells

RNA recovery, cDNA synthesis, and PCR amplification were carried out as previously described [37, 68, 69] with a few minor modifications. 45μL of RLT lysis buffer supplemented with β-ME was added to previously lysed cells for a total volume of 75μL RLT, and RNA was column-purified with RNeasy Micro Kit (Qiagen, Venlo, Netherlands). 14μL of RNA was used directly in a 20μL cDNA synthesis reaction using the High-Capacity cDNA Reverse Transcription kit according to manufacturer’s instructions (Applied Biosystems / Life Technologies, Grand Island, NY). IGH, IGK, and IGL gene transcripts were then amplified independently from cDNA using first and second round primers followed by nested PCR previously published (S2–S4 Tables) [37, 69, 70]. First and second round PCR were done using Phusion High Fidelity DNA polymerase according to manufacturer’s protocol. The first round PCR included 2 mins at 94C followed by 50 cycles of 94°C 10s, 55°C 30s, 72°C 30s with a final extension at 72°C 5mins. 3μl of primary PCR product was used in the nested second round PCR, which included 2 min at 94°C followed by 50 cycles of 90°C 30 s, 72°C 30 s, 72°C 5 min, and cooling at 4°C 15min. PCR products were evaluated on 1.2% flash gels (Lonza, Rockland, ME) and band sizes at ~450–500 bp were purified via Qiaquick purification columns (Qiagen, Venlo, Netherlands) or via Agencourt AM Pure XP beads (Beckman Coulter, Brea, CA). A third and final PCR using Accuprime pfx (Life Technologies, Carlsbad, CA) was performed to add MiSeq adaptors that were used to prime direct amplicon sequencing. The PCR program was initiated with 5 min at 95°C followed by 10 cycles of 95°C 15 s, 55°C 30 s, 68° 30 s, 68°C 5 min, and cooling at 4°C 15min. Third round PCR product was sent for Sanger sequencing (Genewiz, Plainfield, NJ or SeattleBiomed, Seattle, WA). If sequencing reads were unclear, second round nested PCR products were TOPO cloned following manufacturer’s instructions (Life technologies, Carlsbad, CA) and then sent for Sanger sequencing. Paired heavy and light chain sequences were matched against both the human genes via IGMT/V-quest and against the rhesus macaque genes via a customized IgBlast database search using an IGH/IGK/IGL database created and described previously [28, 37, 69, 71–73]. Further analysis was conducted with Geneious Alignment software (cite PMID: 22543367). Antibody sequences can be found in Genbank database with accession numbers MF346735,MF346736, MF346737, MF346738, MF346739, MF346740, MF346741, MF346742, MF346743, MF346744, MF346745, MF346746, MF346747, MF346748, MF346749, MF346750, MF346751, MF346752, MF346753, MF346754, MF346755, MF346756, MF346757, MF346758.

### Heavy and light chain sequencing from bulk B cells

RNA was recovered from 1,000–100,000 sorted B cells (CD19^+^ / IgG^+^ / CD27^+/-^) from PBMCs using the flow staining protocol described above. RNA was recovered using the RNeasy Micro Kit, as described above. All 14μL of recovered RNA were ran through a speed vacuum for 3 min then used directly in the 12μL cDNA synthesis reaction using the Superscript III First-Strand Synthesis SuperMix kit according to manufacturer’s instructions (Invitrogen / Life Technologies, Grand Island, NY). The RT program was initiated with a pre-warmed PCR for 5 min at 65°C followed by ice for 1 min and addition of enzyme mix, then by 1 cycle of 50°C 50 min, 25°C 10 min, 50°C 50 min, and termination at 85°C 5 min followed by chilling on ice. IGH, IGK, and IGL gene transcripts were amplified independently from cDNA using adaptor PCR described above. The adaptor PCR step was performed on 5μL diluted cDNA using Accuprime pfx according to manufacturer’s protocol (Life Technologies, Carlsbad, CA). Adaptor PCR product was purified via Agencourt AM Pure XP beads as described above for 35–50 cycles (Beckman Coulter, Brea, CA). The index PCR step was performed on 5μL cleaned adaptor round PCR product using the Nextera XT DNA Library Preparation kit (Illumina, San Diego, CA) with the Kapa HiFi DNA polymerase PCR program initiated at 3 min at 95C followed by 15–25 cycles of 98°C 20 s, 55°C 15 s, 72° 15 s, 72°C 1 min, and cooling at 4 C 15min (Kapa Biosystems, Wilmington, MA). Index PCR product band size of 600 – 650bp was confirmed on a gel and purified via Agencourt AM Pure XP beads as described above (Beckman Coulter, Brea, CA).

Each library was diluted to 10nM, quantitated with Qubit and Bioanalyzer, and ran on Illumina HiSeq 2500 (Illumina, San Diego, CA) at 2 x 300 with the v3 25M kit at the Genomics Core at the Fred Hutchison Cancer Research Center (FHCRC). Sequencing of multiple libraries (limit of up to 20 per chip) were performed during every sequencing run and a single library was never sequenced alone, thus the error PCR rate was the same for all libraries per chip. Additionally, during each sequencing run, an internal control was included to ensure the proper performance of the sequencer. Illumina data was processed, as previously described (PMID: 27525066). Briefly, raw data obtained from the forward and reverse MiSeq reads were merged to reconstruct the amplicon with FLASH (ver. 1.2.11) (PMID: 21903629). The resulting amplicon sets were filtered to select only sequences containing the amplification primers (a procedure during which the primer sequences themselves were removed) using cutadapt (ver. 1.14); amplicons containing low-confidence base calls (N’s) were then removed from the set, and deduplicated using FASTX-toolkit (ver. 0.0.14) (MARTIN, Marcel. Cutadapt removes adapter sequences from high-throughput sequencing reads. EMBnet.journal, [S.l.], v. 17, n. 1, p. pp. 10–12, may. 2011. ISSN 2226-6089). Samples were annotated using a local IgBLAST (ver. 1.6.1) [74] installation equipped with a custom database of previously published rhesus macaque gene segments (described here [28]. The resulting annotated datasets contained assignments for the most likely matches to the database V/D/J segments and identified the CDR3 sequences for each processed amplicon. Amplicons representing productively-rearranged immunoglobulin sequences were then clustered based on three parameters: 1) V-family assignment, 2) J-family assignment, 3) CDR3 amino acid sequence. Amplicons that shared the CDR3 amino acid sequence but disagreed on the V- or J-family assignment were labeled as "chimera" and filtered out from further analysis. Only clusters containing 5 or more members were considered in further analysis. Cluster V-gene segment assignment was made based on the most abundant assignment found within each cluster; these assignments were then compiled into a gene counts table for each dataset for subsequent analysis with Bioconductor R package edgeR (ver. 3.16.5) [75]. Data sets representing WT and NLGS3-core immunizations were analyzed separately, and log fold-change (logFC) in enrichment for each gene segment as well as the associated false discovery rate (FDR) were used to identify changes associated with the immunization regimens. Structures of clustered sequence populations were analyzed using the R-package *alakazam* (PMID: 26069265) by calculating the values for the general Hill diversity index (encompassing representations of Shannon’s entropy, evenness, etc.).

### Enzyme-Linked Immunosorbent Assay (ELISA)

ELISA assays were carried out using the following antigens: 426c. WT gp140, 426c.NLGS-1 gp140, 426c.NLGS-2 gp140, 426c.NLGS-3 gp140, 426c.NLGS-3.MKE Core gp140, 426c.NLGS-3.MKE CD4bs-KO gp140, 426c.WT.MKE gp120, 426c.NLGS-3.MKE gp120, and HXB2 gp41. 50ng of rEnv was adsorbed onto each well of 96-well or 384-well MaxiSorp ELISA plates (Sigma, St. Louis, MO) overnight in 0.1M NaHCO3, pH 9.4–9.6 at RT. Plates were blocked using dilution buffer, a solution of Millipore H2O, 1X PBS, 10% non-fat dry milk, and 0.03% Tween-20 (Sigma, St. Louis, MO) for 1 h at 37°C. All supernatant from sorted single B cells growing on feeder cells was diluted 1:5 and 1:10 in complete RPMI and incubated for 1 h at 37°C. Serum was diluted 1:10 in dilution buffer and serially titrated 3-fold. Antibodies were diluted to 100μg/mL in dilution buffer and titrated 3-fold. A 3-fold titration of germline and mature VRCO1 was performed as a positive control. Bound antibodies were detected at 37°C for 1h with goat-anti-human-IgG (H+L) HRP conjugate (Invitrogen, Grand Island, NY), diluted 1:6,000 with dilution buffer. Plates were developed with 30μl (384-well) or 50μl (96-well) SureBlue Reserve TMB Microwell Peroxidase Substrate (KPL, Gaithersburg, MD), stopped with an equal amount of 1N H2SO4. Absorption at 450nm was read on a Spectramax spectrophotometer (Molecular Devices, Sunnyvale, CA). ELISA positive wells were above background of 0.2nm at a 1:5 dilution. Assays were performed in triplicate.

### Expression and purification of monoclonal antibodies

Paired IGH and IGL/IGK sequences from isolated single B cells were fabricated into gBlocks (IDT, Coralville, IA) containing flanking regions with corresponding enzyme sites for vector-ligation. gBlocks were digested with the appropriate restriction enzymes EcoRI, NheI (γ), Bsi-WI (κ), XhoI (λ) (NEB, Ipswich, MA) and purified with the Qiagen PCR Purification Kit (Qiagen, Venlo, Netherlands). Ligation was performed for 1–2 hours in a total volume of 10–20μL with 4U T4 DNA Ligase (Invitrogen/Life Technologies, Grand Island, NY), 15ng digested and purified DNA, and 70ng linearized pt1-732 gL γ, pt1-695 κ, pt1-341 λ vectors. Chemically competent DH5α T1 E. coli cells were transformed with 2.5μL of ligation product. Single colonies were expanded for 16h at 37°C in 4mL LB Broth containing 1 μg/mL ampicillin. Plasmid DNA was purified via Qiagen Qiaprep Mini-Prep kit (Qiagen, Venlo, Netherlands) and eluted with 30μL EB Buffer. Plasmid DNA was quantified using a NanoDrop spectrometer (Thermo Fisher Scientific, Waltham, MA) and sequenced by the Sanger sequencing method (Genewiz, South Plainfield, NJ) using a pTT3 5’ forward primer. Plasmids containing confirmed sequences were expanded for 12h at 37°C in 100mL LB Broth containing 1μg/mL ampicillin. Plasmid DNA was purified with a HiSpeed Plasmid Maxi Kit (Qiagen, Venlo, Netherlands), eluted with 50μL TE Buffer, and filtered with a 0.2μM filter before co-transfection.

IgGs were produced by transient co-transfection of two plasmids: one expressing the IGH and the other the IGL/IGK. Briefly, 12.5μg of IGH plasmid DNA and 12.5μg of IGL/IGK plasmid DNA were incubated at RT for 15 m with 293F transfection reagent (EMD Millipore, Temecula, CA) in 1x PBS prior to addition to 293F cells (HEK-293F suspension cells from the American Type Culture Collection (ATCC)), these are a variation of human embryonic kidney cells derived from an unknown original patient)) at 1 million cells/mL in 50mL of FreeStyle 293 Expression Media (Life Technologies, Grand Island, NY). After 4–6 days incubation, the cell supernatants were centrifuged at 6,000rpm for 10min and the clarified supernatant was filtered through a 0.2μM filter (Millipore, Billerica, MA) before loading onto a pre-rinsed Protein-A/G agarose resin column (Thermo Fisher Scientific, Waltham, MA). After washing the agarose beads with 10x column volumes of 1x PBS, IgG was eluted from the column with 0.1 M citric acid (pH 3) in 1mL fractions into tubes containing 100μL 1M Tris-HCl (pH 9). Fractions with high IgG content were pooled and buffer exchanged into 1x PBS using Amicon Ultra-4 centrifugal units with 30kDa membrane cutoffs (Millipore, Billerica, MA). IgG concentrations were determined using a NanoDrop spectrometer (Thermo Fisher Scientific, Waltham, MA) and antibody size confirmed via SDS-PAGE/Western Blot expression.

### Competition Biolayer Interferometry (BLI)

BLI was performed on purified biotinylated IgG using an Octet Red instrument (ForteBio, Inc., Menlo Park, CA). Antibodies were biotinylated in water using the EZ-Link (NHS-PEG4-Biotin) Kit according to manufacturer’s instructions (Thermo Fisher Scientific, Waltham, MA). Biotinylated antibodies were buffer exchanged with 1x PBS and purified via Amicon Ultra-4 centrifugal units with 30kDa membrane cutoffs (Millipore, Billerica, MA). IgG concentrations were determined using a NanoDrop spectrometer (Thermo Fisher Scientific, Waltham, MA). For these assays, gp140 trimeric Env forms were resuspended at 80nM in 1x Kinetics Buffer. Streptavidin (SA) biosensors (ForteBio, Inc., Menlo Park, CA) were activated by immersion into 1x Kinetics Buffer (1x PBS, 0.1% BSA, 0.02% Tween-20, 0.005% NaN_3_) for 10m. Biotinylated IgGs (at 10μg/mL in 1x KB) were immobilized on SA biosensors for 300s, and then biosensors were re-immersed in 1x Kinetics Buffer for 60s to establish a ‘baseline’. Biosensors were then immersed into wells containing NLGS-3 Core rEnv gp140 or NLGS-3 Core rEnv gp140 previously incubated with saturating concentrations (160nM in 1x Kinetics Buffer) of mature VRC01, mature b12, or CD4-IgG [(CD4-IgG obtained through the National Institutes of Health (NIH) AIDS Research and Reference Reagent Program, Division of AIDS, National Institute of Allergy and Infectious Diseases, NIH (cat. no. 11780; contributors: Progenics Pharmaceuticals, Inc)]. After an association phase of 300s, SA biosensors were re-immersed into wells containing only 1x Kinetics Buffer for dissociation for 600s. Binding shift (nm) was determined by alignment to baseline, interstep correction to dissociation, and final processing with Savitsky-Golay Filtering.

### Virus neutralization assays

Heat-inactivated serum from immunized animals, supernatants from Env-specific sorted B cells (see above), or monoclonal antibodies (MAbs), were tested for neutralizing activity using the TZM-bl (also known as JC53BL-13 cells, a Henrietta Lacks (HeLa) cell clone engineered to be CXCR4-positive was obtained from the Center for AIDS Reagents (CFAR)) cell line-based neutralization assay, as previously described [31, 76]. Briefly, MAbs (starting concentration 50μg/mL or 200μg/mL), B cell supernatant (starting at 1:2 dilution), or sera (starting at 1:10) were serially diluted 3-fold for at a final volume of 30μL. 30μL of pseudovirus, previously determined to result in ~2 x 10^5^ luciferase units per well, was added to each well for 90 min at 37°C. 50μL of the pre-incubated mixture was added to TZM-bl cells, previously incubated with polybrene for 30min at 37°C. 72 hours later, the media were removed and Steady-Glo Luciferase reagent (Promega, Madison, WI) was added.

### Statistical analysis

All assays were performed in duplicate or triplicate as indicated. Significant difference were assessed by ANOVA via Prism v6 software. Bioinformatics analysis significant differences were calculated in various R packages as indicated above. Respective p-values and FDR values are indicated in figure legends.

## Supporting information

S1 Fig Immunization schedule. Rhesus macaques were immunized twice with DNA and twice with DNA/ recombinant protein. Group 1 (N = 4) received 426c WT gp140 and group 2 (N = 4) received 426c NLGS-3 Core gp140. The timing of each immunization and the timing of sample collection are indicated.
(TIF)

S2 Fig CD4-binding site specificities of immune serum antibodies. Env-binding experiments were performed with serum collected prior to the initiation of immunizations (Pre-immunization) and following Post DNA/Protein Boost 1, and Post DNA/Protein Boost 2 immunizations with WT (A) and NLGS-3 Core (B) immunogens. The recombinant Env proteins used during these experiments include the autologous immunogen to the vaccine and their CD4bs-KO forms. The average EC50 and standard deviations of serum reactivities of four animals per immunization group are shown. Standard deviations indicate: * *indicates a p value < 0*.*01*, ** *indicates a p value < 0*.*001*, *** *indicates a p value < 0*.*0001*, **** *indicates a p value < 0*.*00001*.
(TIF)

S3 Fig Relative changes in VH/VL frequencies in peripheral B cells. Relative changes for WT sequencing data sets are presented as the Log in fold change (LogFC) for IGH (A), IGK (B), and IGL (C) V alleles between pre-immunization and following the last DNA alone (red triangles) or the last DNA/protein boost immunization (blue squares). Positive LogFC with FDRs < 0.05 are those present above the dashed lines. False discovery rate (FDR, p value adjusted for multiple testing) and presence in 2 or more animals per group). IGHV (A), IGKV (B) and IGLV (C) alleles with a significant LogFC are shown.
(TIF)

S4 Fig Population structure of the pre-bleed time point (-2 weeks) PBMC-derived NGS sequence sets following clustering analysis. Sequence sets from each animal are represented using Hill’s diversity curves for (A) WT immunized macaques and (B) NLGS-3 Core immunized macaques, separated by chain: IgG, IgK, and IgL. *D(0)* values are shown on the *y-axis*.
(TIF)

S5 Fig Population structure of the post DNA prime immunization time point (week 5) PBMC-derived NGS sequence sets following clustering analysis. Sequence sets from each animal are represented using Hill’s diversity curves for (A) WT immunized macaques and (B) NLGS-3 Core immunized macaques, separated by chain: IgG, IgK, and IgL. *D(0)* values are shown on the *y-axis*.
(TIF)

S6 Fig Population structure of the post DNA-Protein second boost immunization time (point week 39) PBMC-derived NGS sequence sets following clustering analysis. Sequence sets from each animal are represented using Hill’s diversity curves for (A) WT immunized macaques and (B) NLGS-3 Core immunized macaques, separated by chain: IgG, IgK, and IgL. *D(0)* values are shown on the *y-axis*.
(TIF)

S7 Fig Population structure of the lymph node derived IgG NGS sequence sets at all time points from all NLGS-3 Core immunized animals following clustering analysis. Sequence sets from each NLGS-3 Core immunized animal are represented using Hill’s diversity curves for IgG separated by weeks post immunization: -2, 5, and 39. *D(0)* values are shown on the *y-axis*.
(TIF)

S8 Fig Principal component analysis (PCA). PCA clusters variables within large, complex data sets by the sources of variation. For WT (A) sequence data sets group by time point, indicating that the changes in gene frequency in the NGS data sets are due to treatment (i.e., vaccination time point). In contrast, the NLGS-3 Core (B) sequence sets group by animal and not time point, indicating that vaccination did not drive statistically significant changes in the NGS sequence sets over time.
(TIF)

S9 Fig Isolation and characterization of single B cells from the blood of NLGS-3 Core immunized animals. B cells were isolated from PBMC at the end of immunization from the four animals immunized with the NLGS-3 Core immunogen. The number of IgG+ B cells sorted, the frequency of B cells sorted, the percent of B cells displaying neutralizing activity against the NLGS-3 virus, and the number of neutralizing MAbs isolated from each animal are indicated.
(TIF)

S10 Fig IGHV characteristics of non-neutralizing Abs. VH genes were amplified and sequenced from individual B cells isolated from PBMCs of NLGS-3 Core-immunized animals at the end of the immunization. Percent occurrence in the NGS data sets of the annotated segment allele for the isolated single cell VH gene is indicated for the PBMC, LN, and BM at the end of immunization. N/A indicates percent occurrence of the allele was too low to detect.
(TIF)

S11 Fig IGKV / IGLV characteristics of non-neutralizing Abs. VL (κ and λ) genes were amplified and sequenced from individual B cells isolated from PBMCs of NLGS-3 Core-immunized animals at the end of the immunization. Percent occurrence in the NGS data sets of the annotated gene segment allele for the isolated single cell VH gene is indicated for the PBMC, LN, and BM at the end of immunization. N/A indicates percent occurrence of the allele was too low to detect.
(TIF)

S12 Fig Characteristics of neutralizing antibodies. The antibody, the animal each antibody was isolated from, their allelic derivation, their nucleotide divergence from the germline IGH and IGK or IGL genes and the length of CDRH3 and CDRL3 domains are indicated. The frequency of the corresponding IGH and IGK or IGL genes in the NGS sequence sets from the periphery, lymph nodes and bone marrow are indicated. N/A indicates percent occurrence of the allele was too low to detect. ND indicates this sample was not sequenced by NGS.
(TIF)

S13 Fig Amino acid sequences of the CDR regions of NAbs. (A) CDRH1, CDRH2, and CDRH3 regions of IGHV sequences of NAbs. CDR Regions are compared to their respective germline sequence with differences highlighted in light orange. (B) CDRL1, CDRL2, and CDRL3 regions of IGLV sequences of NAbs. CDR Regions are compared to their respective germline sequence with differences highlighted in light blue. Gold indicates differences in the CDRH3 and CDRL3 regions. *Germline sequences are indicated by grey*.
(TIF)

S1 Table Amino acid sequences of neutralizing antibodies. Heavy (A) and light chain (B) sequences, germline indicated with gray, Kabat numbering is indicated at the top of each column. Antibody nucleotide sequences can be found in Genbank database with accession numbers MF346735 –MF346758.
(TIF)

S2 Table List of IGH primers. Used in first, second (A), and third round (B) PCR of isolated single B cells.
(TIF)

S3 Table List of IGK primers. Used in first, second, and third round PCR of isolated single B cells.
(TIF)

S4 Table List of IGL primers. Used in first, second, and third round PCR of isolated single cells.
(TIF)
