# Supplementary figures and images for: B cell clonal lineage alterations upon recombinant HIV-1 envelope immunization of rhesus macaques
Source: PLoS Pathog. 2018 Jun 22;14(6):e1007120. doi: 10.1371/journal.ppat.1007120 (PMC6033445; doi:10.1371/journal.ppat.1007120)

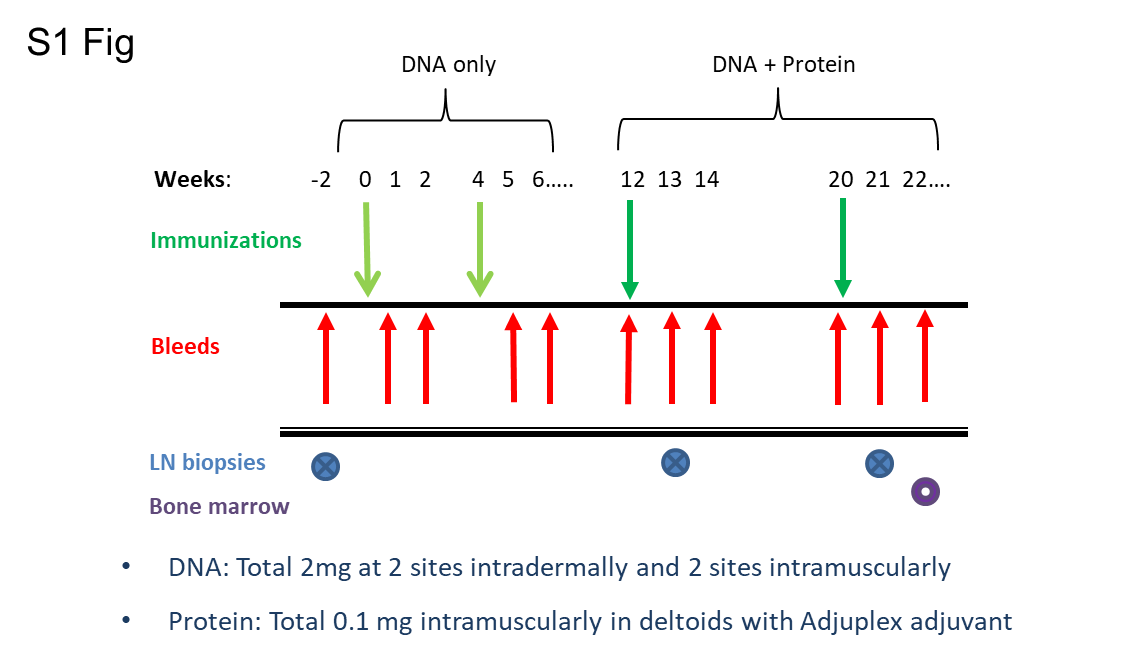

Supplement: S1 Fig — Rhesus macaques were immunized twice with DNA and twice with DNA/ recombinant protein. Group 1 (N = 4) received 426c WT gp140 and group 2 (N = 4) received 426c NLGS-3 Core gp140. The timing of each immunization and the timing of sample collection are indicated. (TIF) [file ppat.1007120.s001.tif]

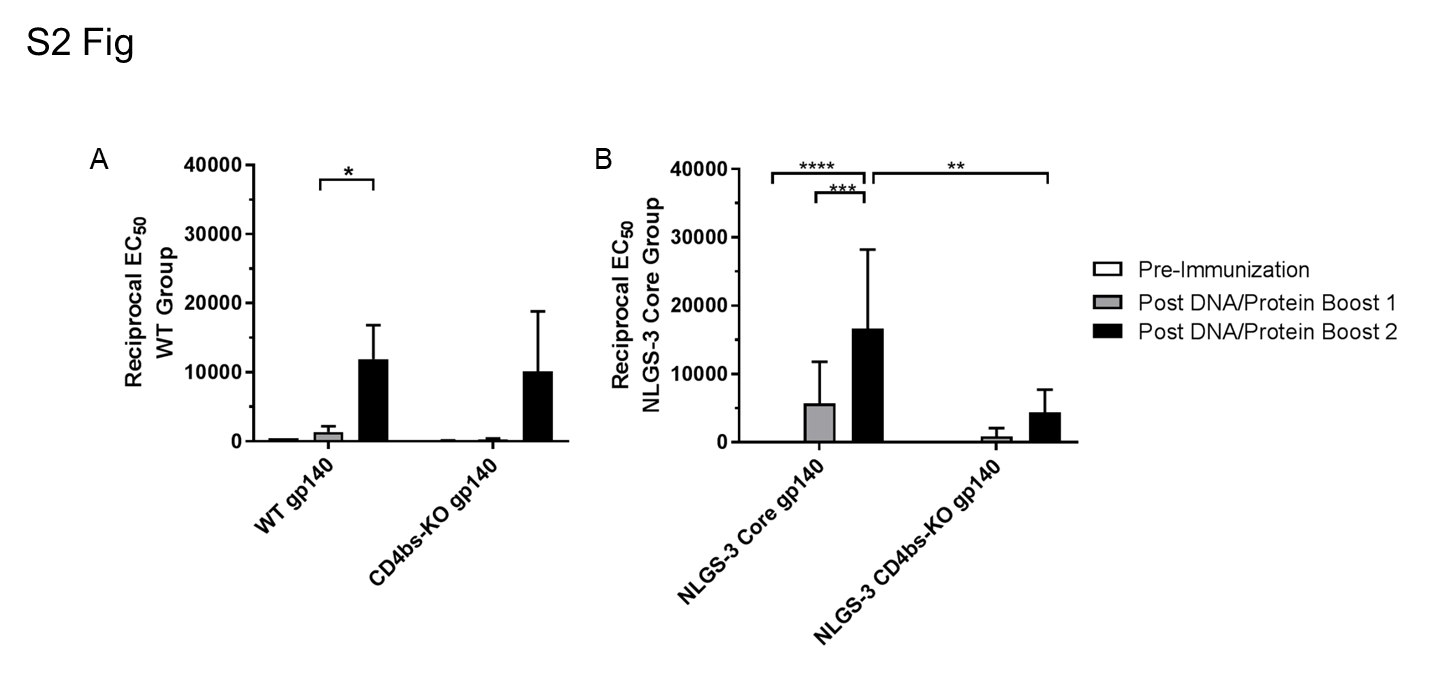

Supplement: S2 Fig — Env-binding experiments were performed with serum collected prior to the initiation of immunizations (Pre-immunization) and following Post DNA/Protein Boost 1, and Post DNA/Protein Boost 2 immunizations with WT (A) and NLGS-3 Core (B) immunogens. The recombinant Env proteins used during these experiments include the autologous immunogen to the vaccine and their CD4bs-KO forms. The average EC50 and standard deviations of serum reactivities of four animals per immunization group are shown. Standard deviations indicate: * indicates a p value < 0.01, ** indicates a p value < 0.001, *** indicates a p value < 0.0001, **** indicates a p value < 0.00001. (TIF) [file ppat.1007120.s002.tif]

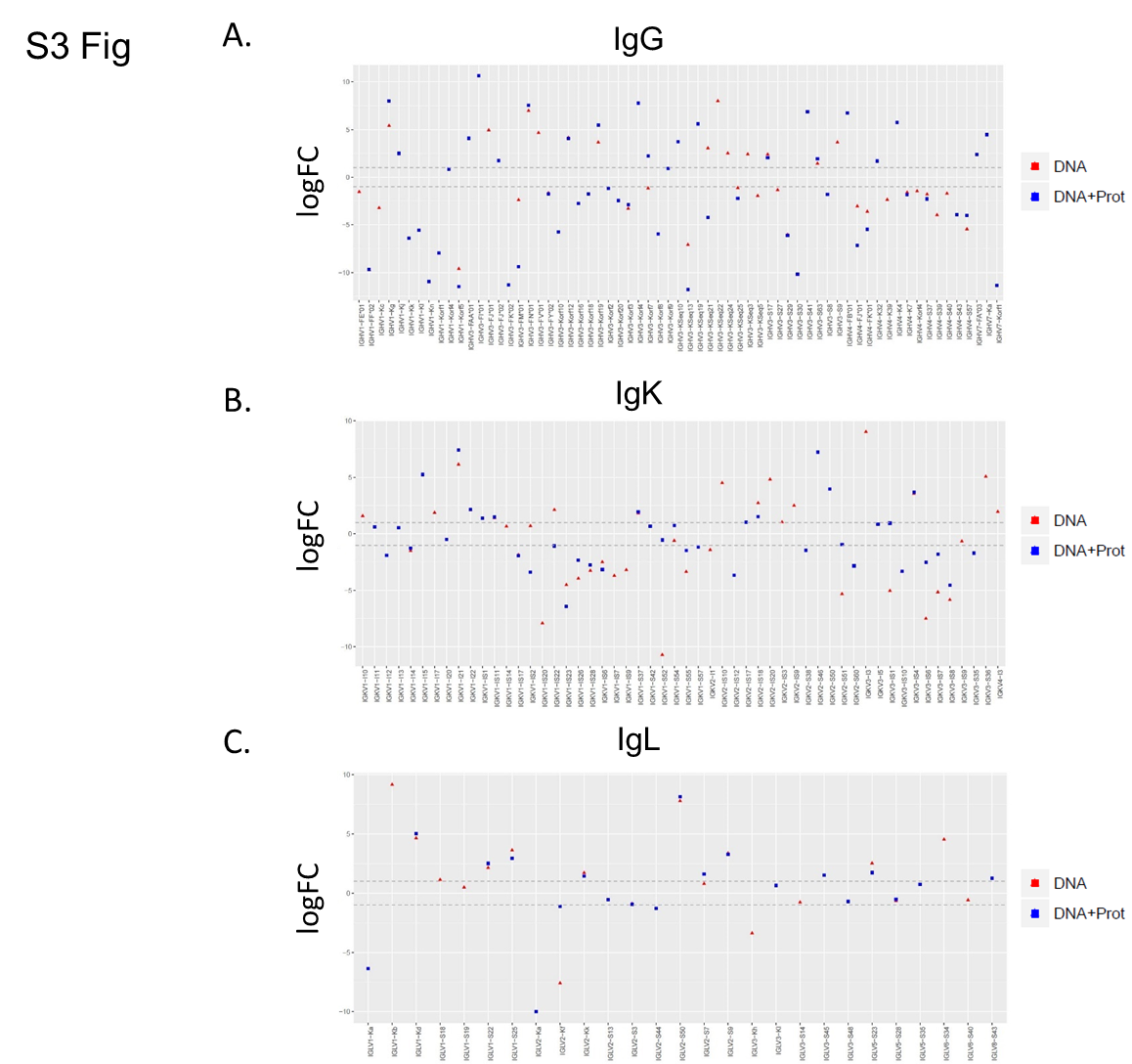

Supplement: S3 Fig — Relative changes for WT sequencing data sets are presented as the Log in fold change (LogFC) for IGH (A), IGK (B), and IGL (C) V alleles between pre-immunization and following the last DNA alone (red triangles) or the last DNA/protein boost immunization (blue squares). Positive LogFC with FDRs < 0.05 are those present above the dashed lines. False discovery rate (FDR, p value adjusted for multiple testing) and presence in 2 or more animals per group). IGHV (A), IGKV (B) and IGLV (C) alleles with a significant LogFC are shown. (TIF) [file ppat.1007120.s003.tif]

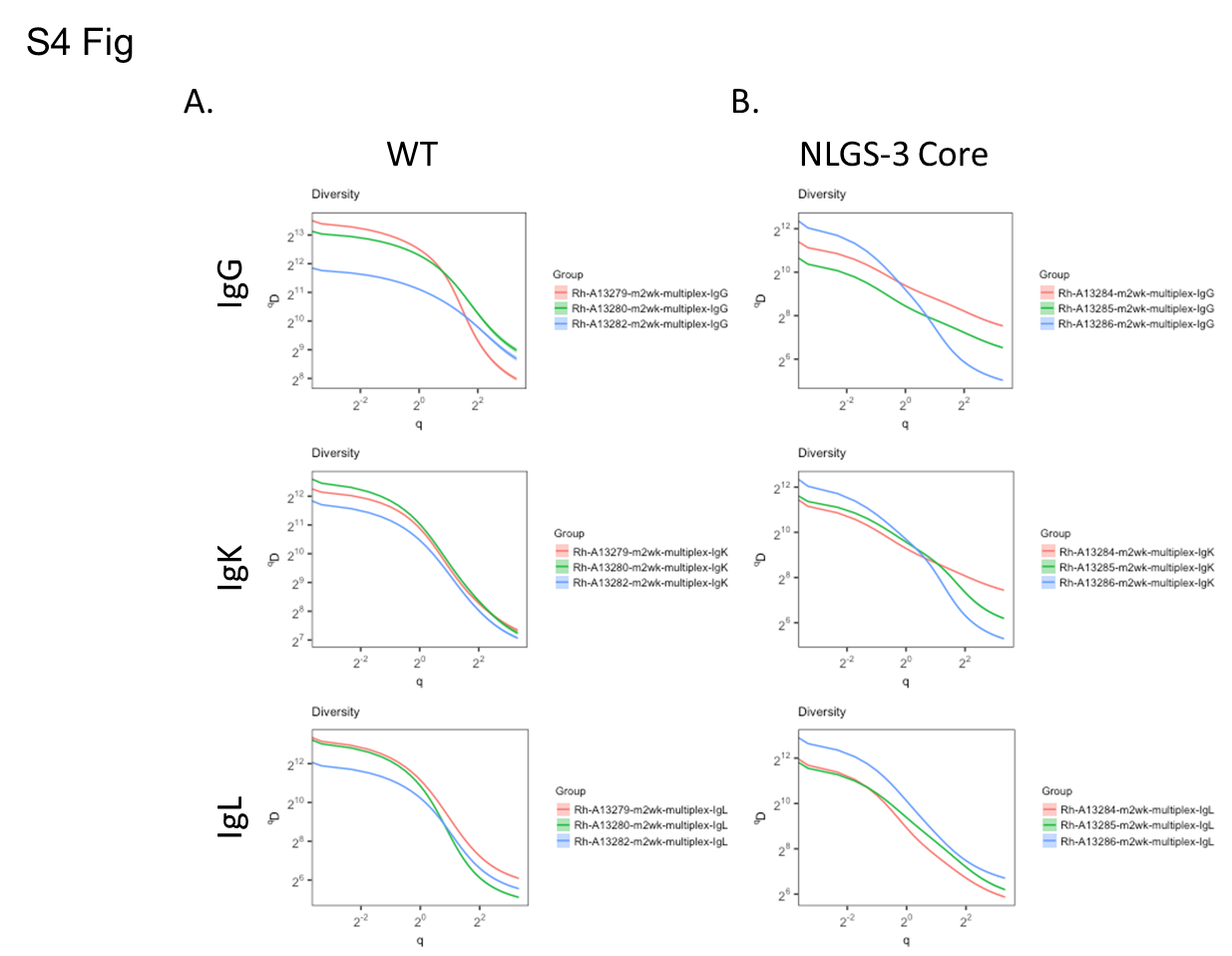

Supplement: S4 Fig — Sequence sets from each animal are represented using Hill’s diversity curves for (A) WT immunized macaques and (B) NLGS-3 Core immunized macaques, separated by chain: IgG, IgK, and IgL. D(0) values are shown on the y-axis. (TIF) [file ppat.1007120.s004.tif]

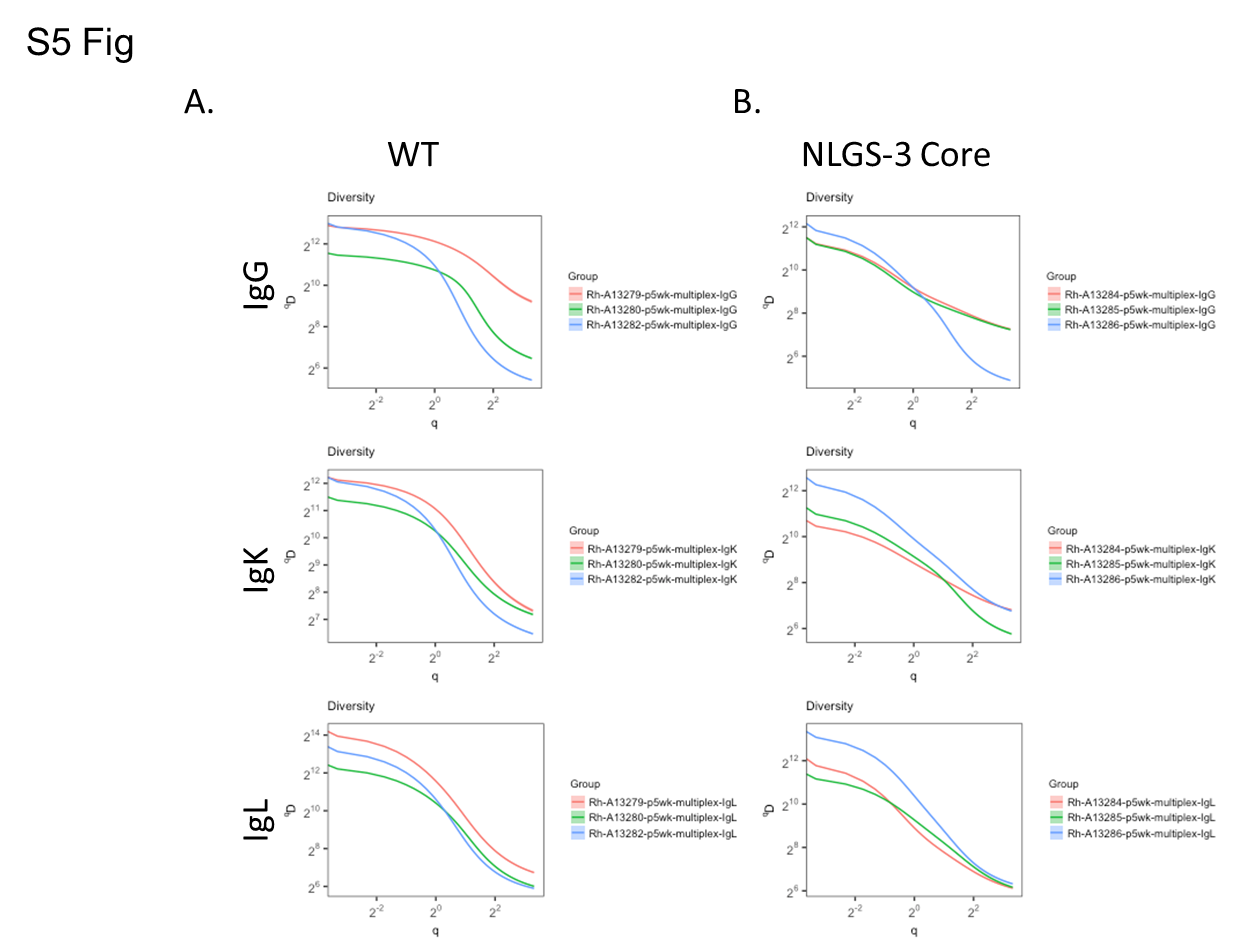

Supplement: S5 Fig — Sequence sets from each animal are represented using Hill’s diversity curves for (A) WT immunized macaques and (B) NLGS-3 Core immunized macaques, separated by chain: IgG, IgK, and IgL. D(0) values are shown on the y-axis. (TIF) [file ppat.1007120.s005.tif]

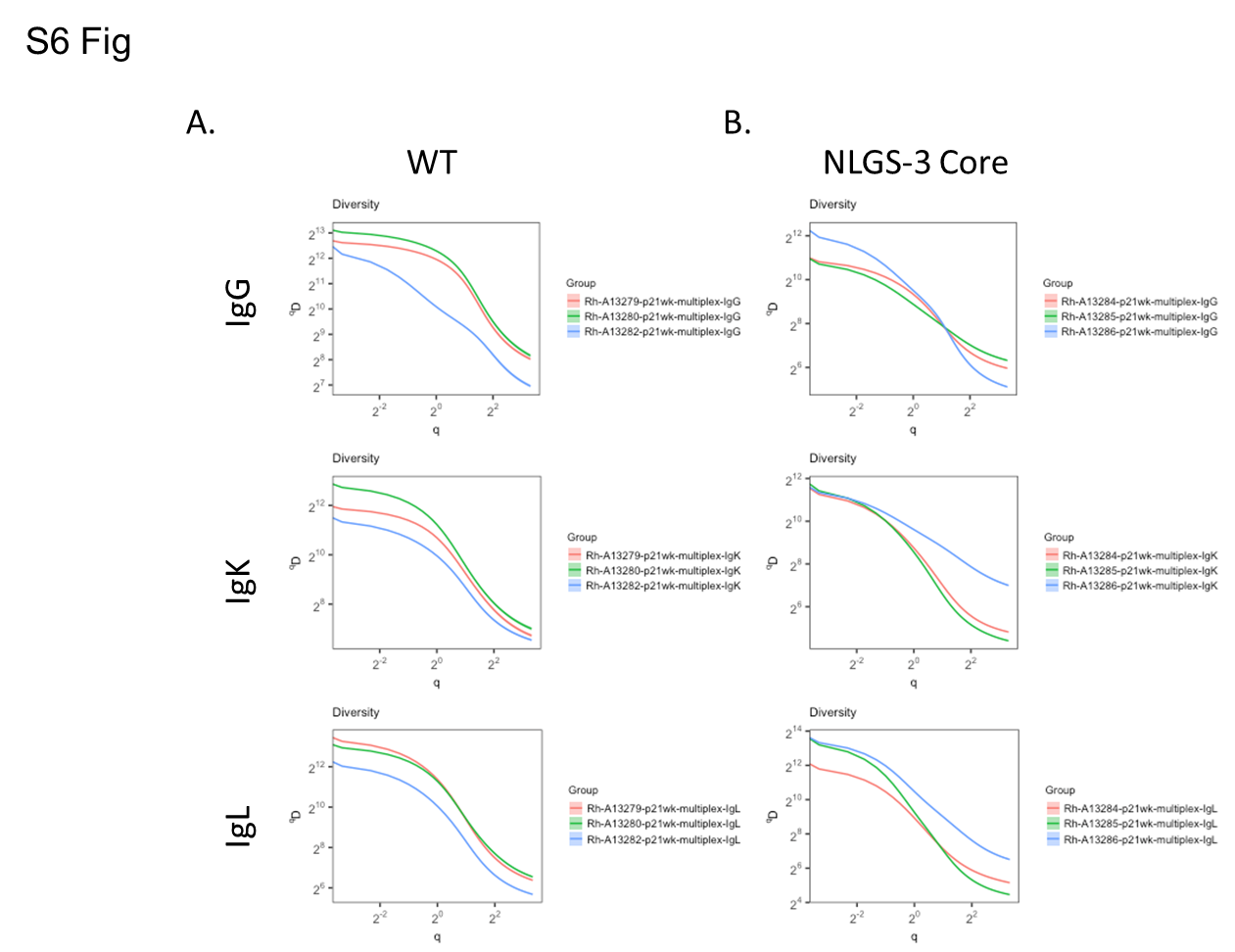

Supplement: S6 Fig — Sequence sets from each animal are represented using Hill’s diversity curves for (A) WT immunized macaques and (B) NLGS-3 Core immunized macaques, separated by chain: IgG, IgK, and IgL. D(0) values are shown on the y-axis. (TIF) [file ppat.1007120.s006.tif]

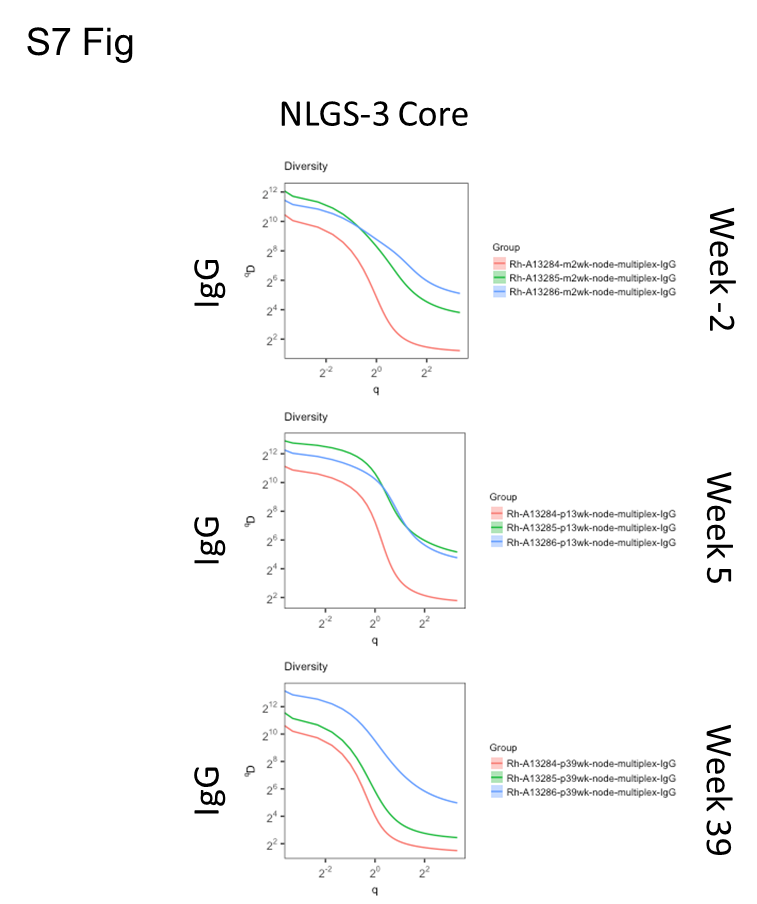

Supplement: S7 Fig — Sequence sets from each NLGS-3 Core immunized animal are represented using Hill’s diversity curves for IgG separated by weeks post immunization: -2, 5, and 39. D(0) values are shown on the y-axis. (TIF) [file ppat.1007120.s007.tif]

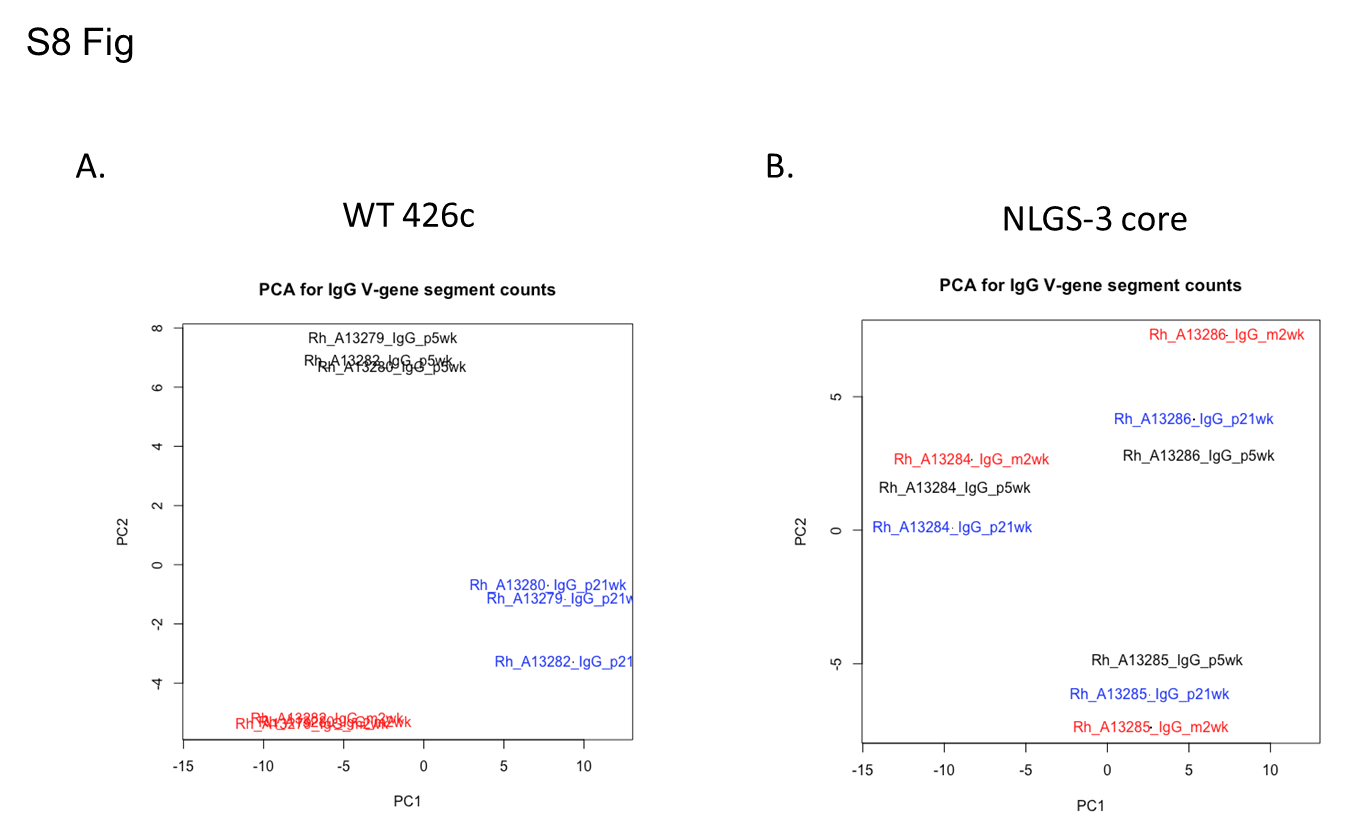

Supplement: S8 Fig — PCA clusters variables within large, complex data sets by the sources of variation. For WT (A) sequence data sets group by time point, indicating that the changes in gene frequency in the NGS data sets are due to treatment (i.e., vaccination time point). In contrast, the NLGS-3 Core (B) sequence sets group by animal and not time point, indicating that vaccination did not drive statistically significant changes in the NGS sequence sets over time. (TIF) [file ppat.1007120.s008.tif]

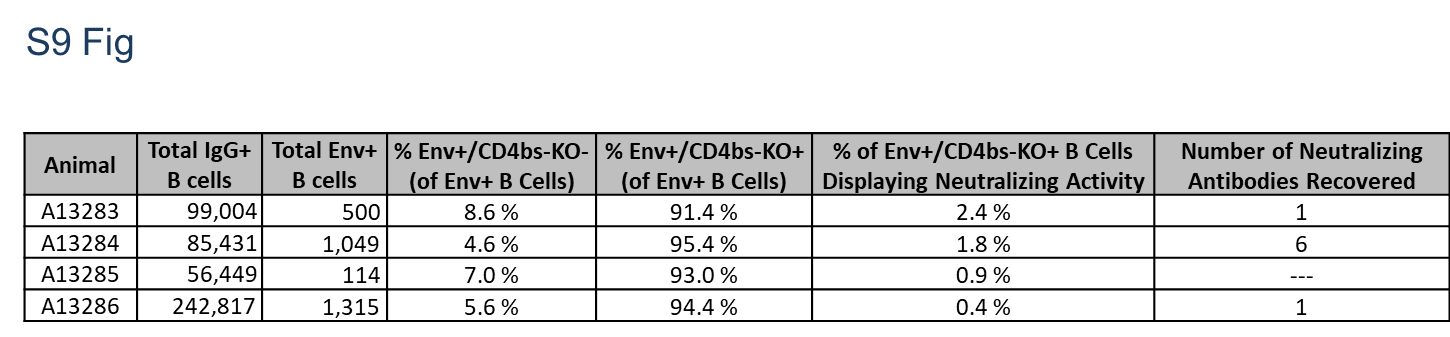

Supplement: S9 Fig — B cells were isolated from PBMC at the end of immunization from the four animals immunized with the NLGS-3 Core immunogen. The number of IgG+ B cells sorted, the frequency of B cells sorted, the percent of B cells displaying neutralizing activity against the NLGS-3 virus, and the number of neutralizing MAbs isolated from each animal are indicated. (TIF) [file ppat.1007120.s009.tif]

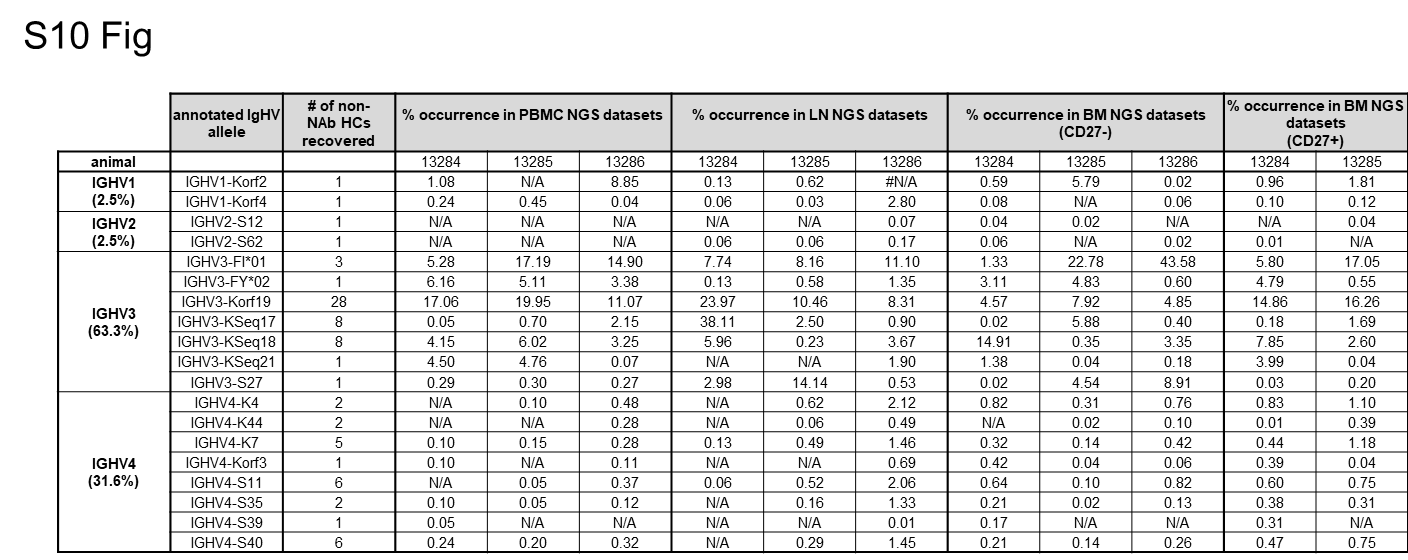

Supplement: S10 Fig — VH genes were amplified and sequenced from individual B cells isolated from PBMCs of NLGS-3 Core-immunized animals at the end of the immunization. Percent occurrence in the NGS data sets of the annotated segment allele for the isolated single cell VH gene is indicated for the PBMC, LN, and BM at the end of immunization. N/A indicates percent occurrence of the allele was too low to detect. (TIF) [file ppat.1007120.s010.tif]

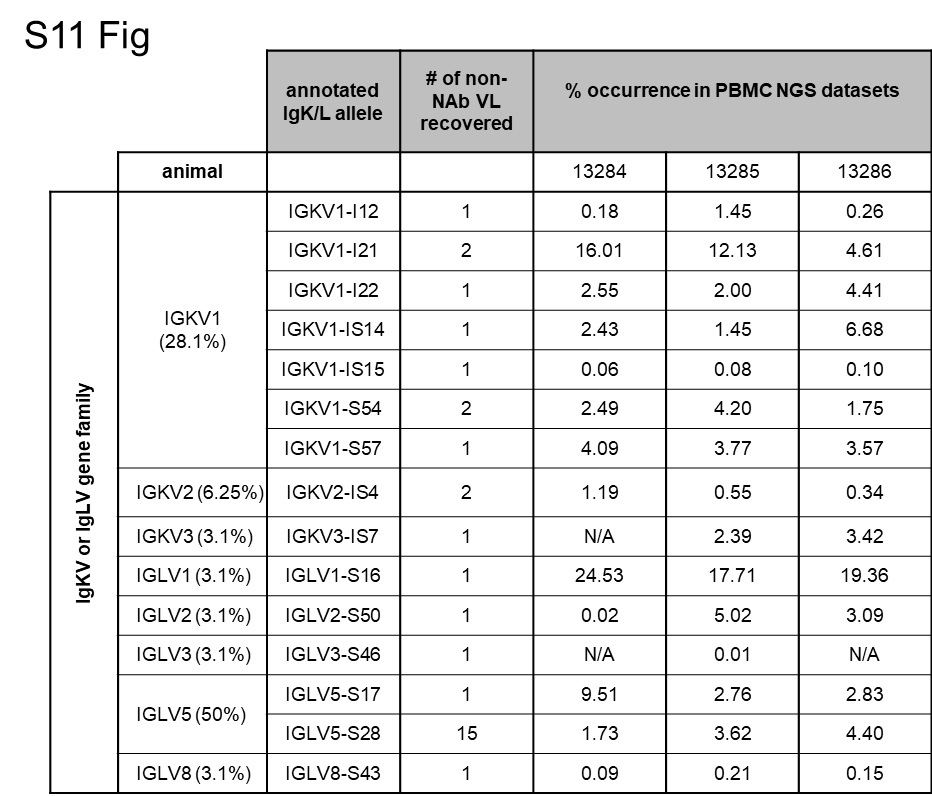

Supplement: S11 Fig — VL (κ and λ) genes were amplified and sequenced from individual B cells isolated from PBMCs of NLGS-3 Core-immunized animals at the end of the immunization. Percent occurrence in the NGS data sets of the annotated gene segment allele for the isolated single cell VH gene is indicated for the PBMC, LN, and BM at the end of immunization. N/A indicates percent occurrence of the allele was too low to detect. (TIF) [file ppat.1007120.s011.tif]

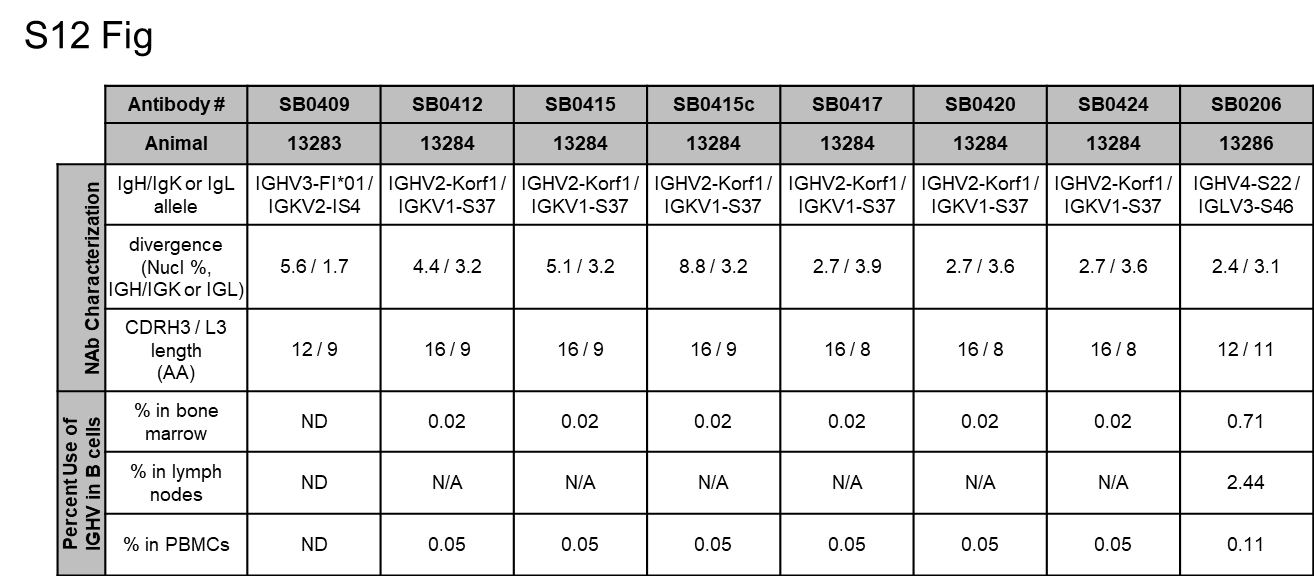

Supplement: S12 Fig — The antibody, the animal each antibody was isolated from, their allelic derivation, their nucleotide divergence from the germline IGH and IGK or IGL genes and the length of CDRH3 and CDRL3 domains are indicated. The frequency of the corresponding IGH and IGK or IGL genes in the NGS sequence sets from the periphery, lymph nodes and bone marrow are indicated. N/A indicates percent occurrence of the allele was too low to detect. ND indicates this sample was not sequenced by NGS. (TIF) [file ppat.1007120.s012.tif]

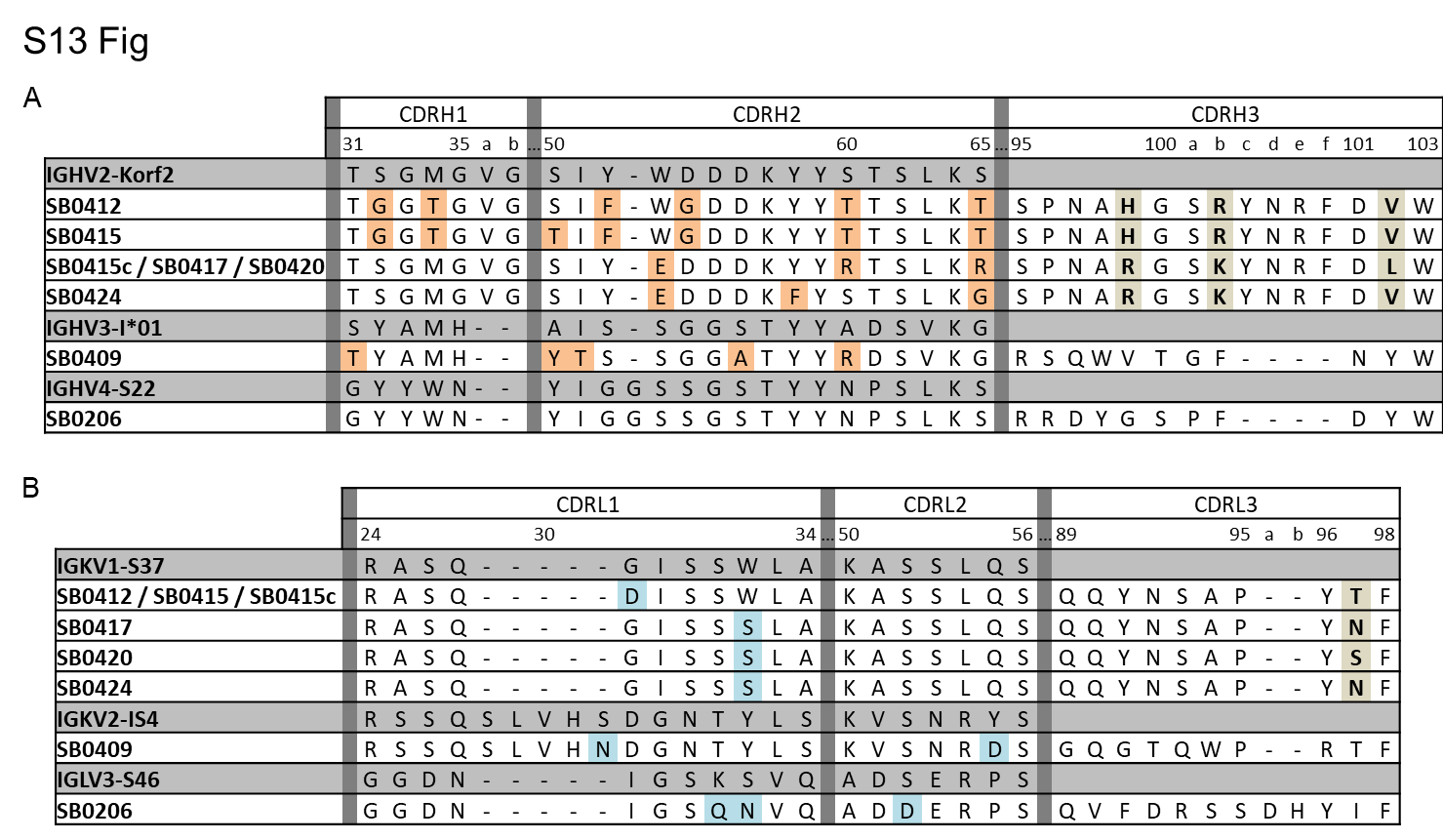

Supplement: S13 Fig — (A) CDRH1, CDRH2, and CDRH3 regions of IGHV sequences of NAbs. CDR Regions are compared to their respective germline sequence with differences highlighted in light orange. (B) CDRL1, CDRL2, and CDRL3 regions of IGLV sequences of NAbs. CDR Regions are compared to their respective germline sequence with differences highlighted in light blue. Gold indicates differences in the CDRH3 and CDRL3 regions. Germline sequences are indicated by grey. (TIF) [file ppat.1007120.s013.tif]

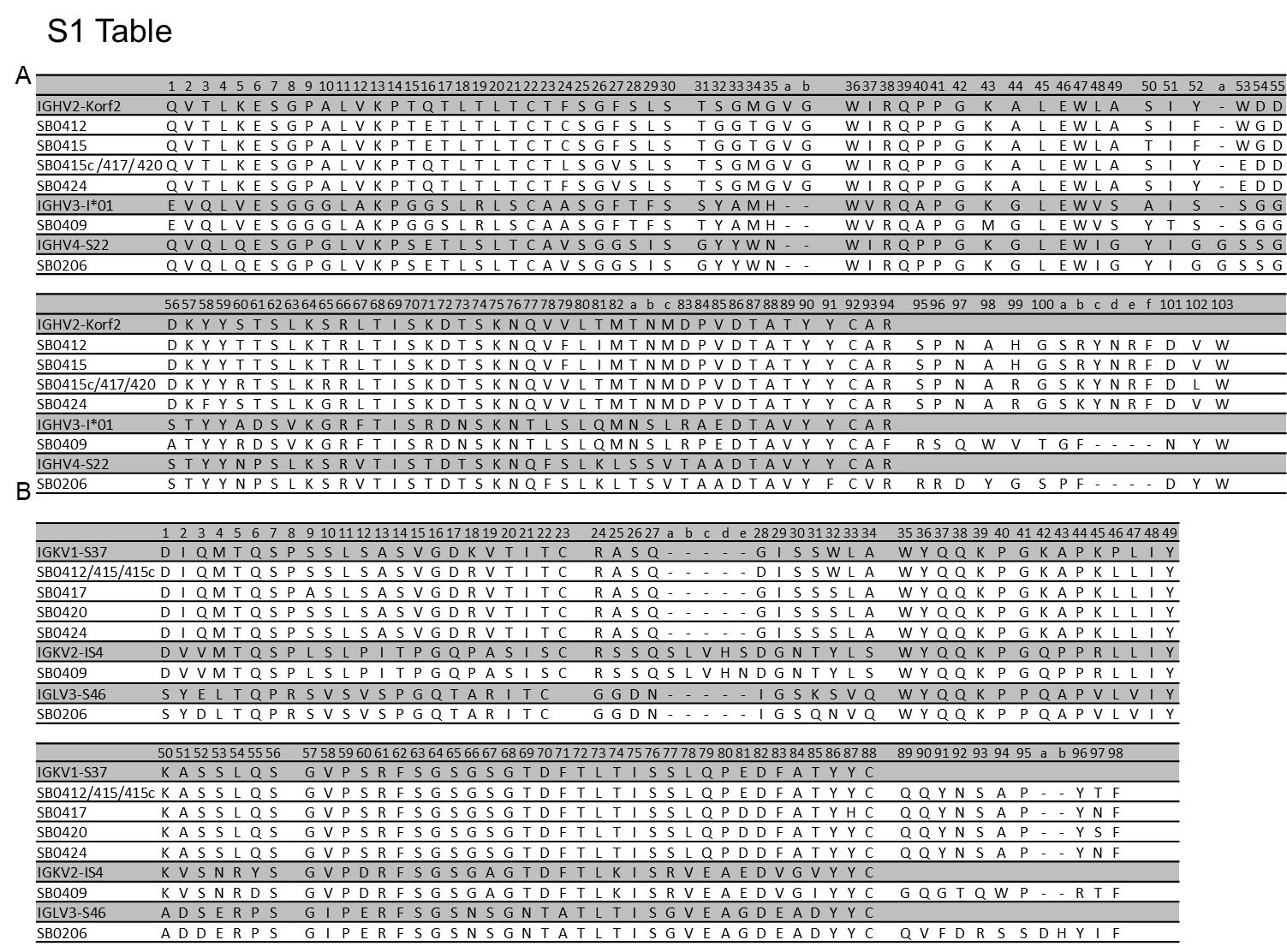

Supplement: S1 Table — Heavy (A) and light chain (B) sequences, germline indicated with gray, Kabat numbering is indicated at the top of each column. Antibody nucleotide sequences can be found in Genbank database with accession numbers MF346735 –MF346758. (TIF) [file ppat.1007120.s014.tif]

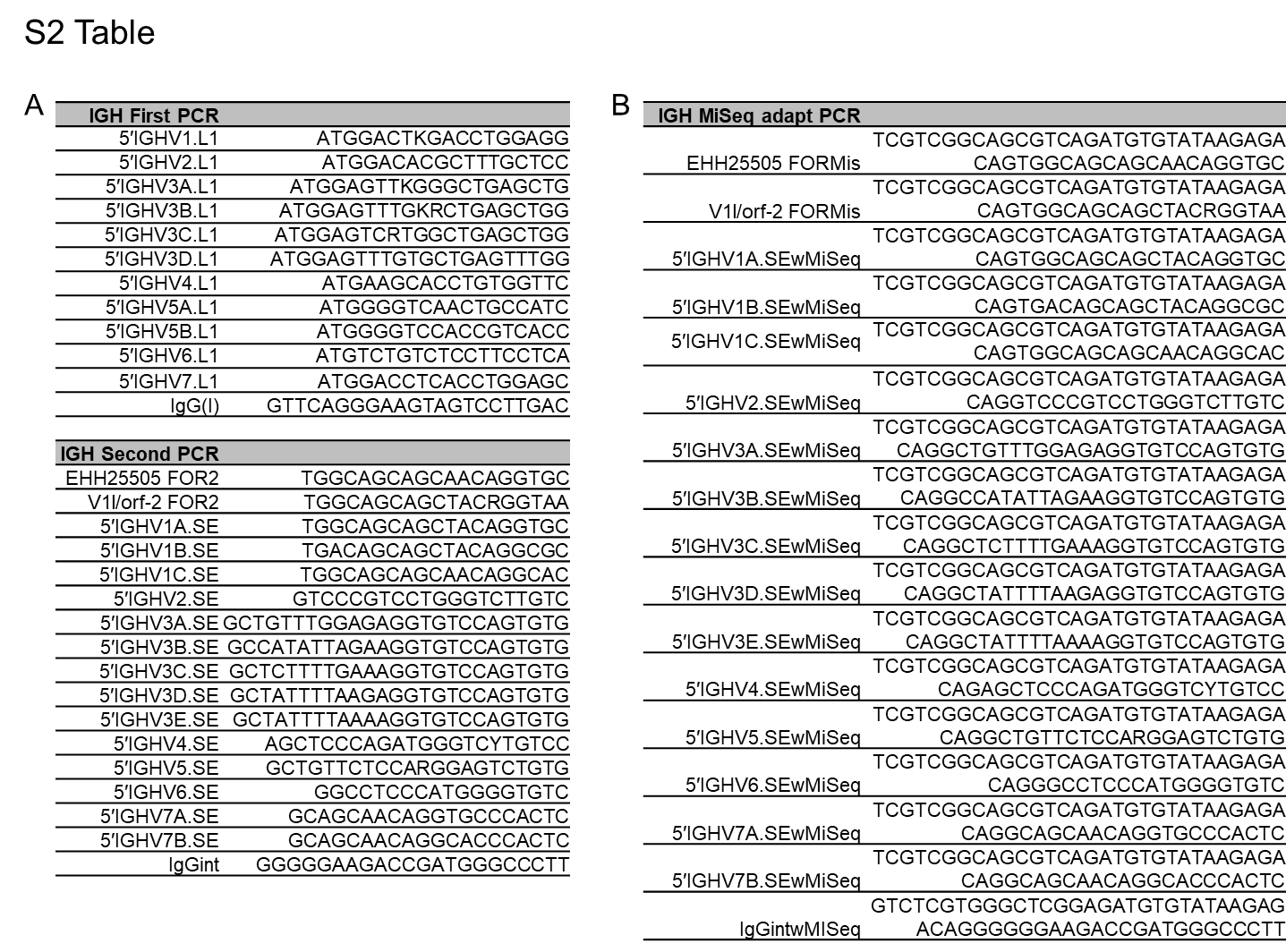

Supplement: S2 Table — Used in first, second (A), and third round (B) PCR of isolated single B cells. (TIF) [file ppat.1007120.s015.tif]

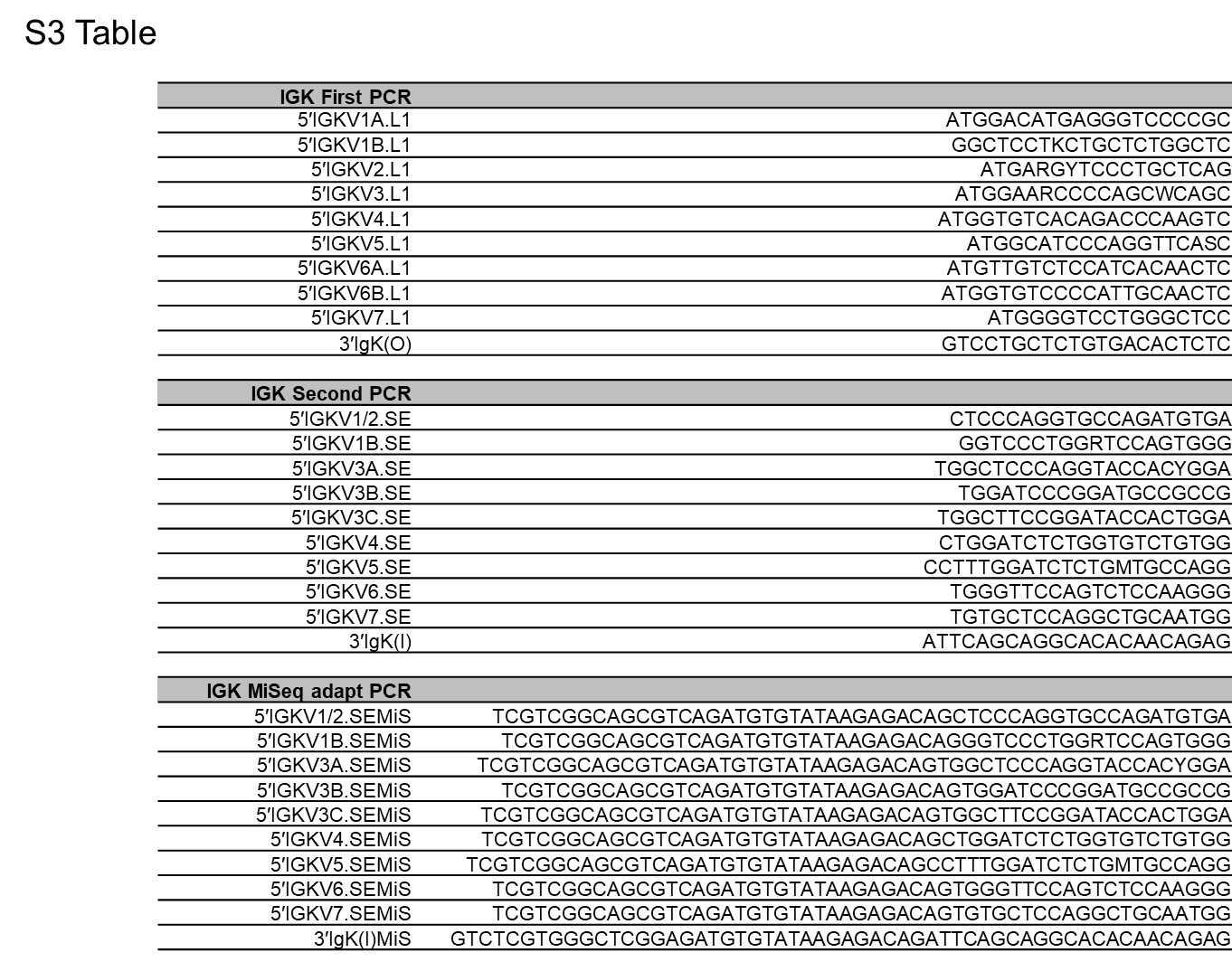

Supplement: S3 Table — Used in first, second, and third round PCR of isolated single B cells. (TIF) [file ppat.1007120.s016.tif]

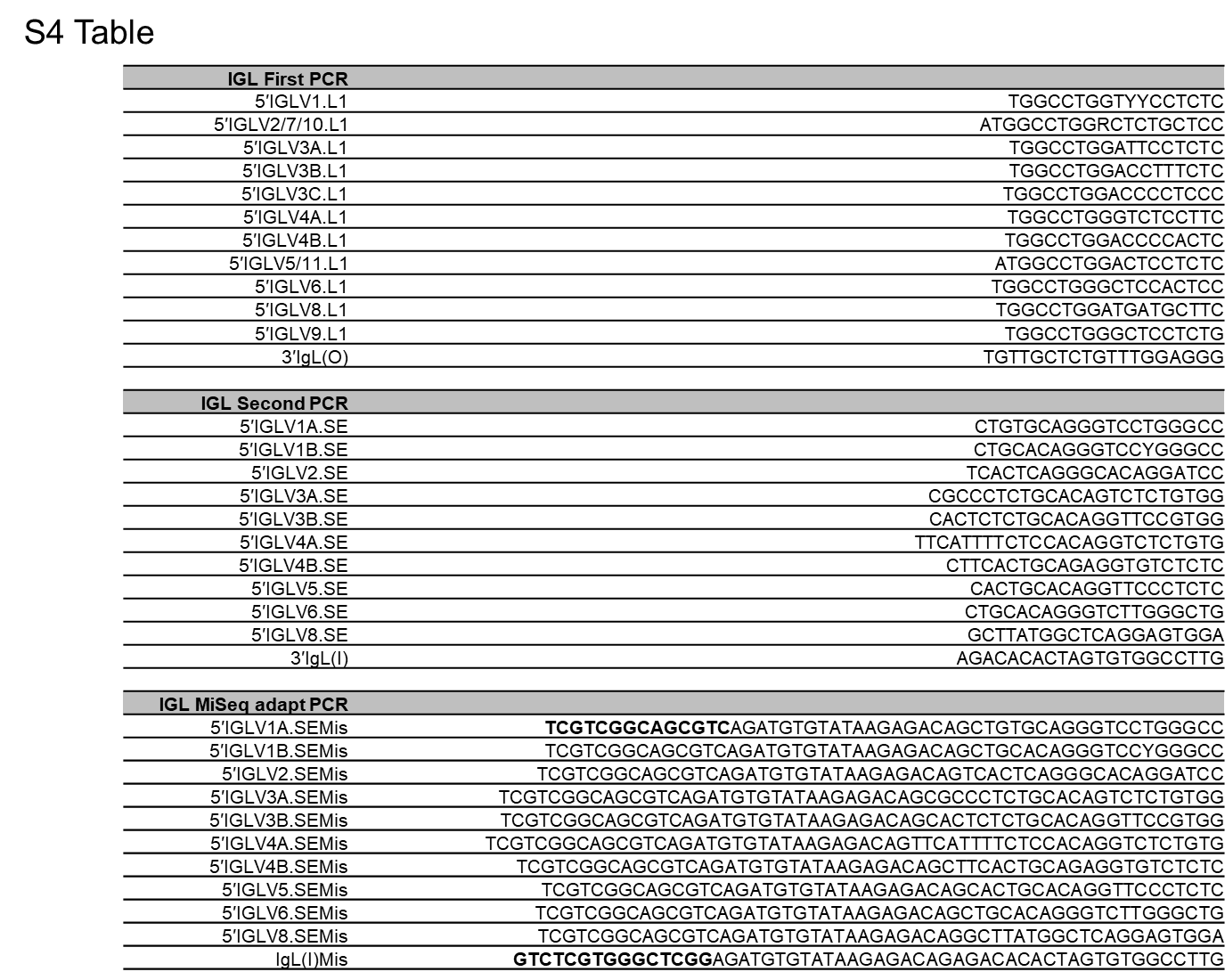

Supplement: S4 Table — Used in first, second, and third round PCR of isolated single cells. (TIF) [file ppat.1007120.s017.tif]
